# Brain-infiltrating CD8 T cells retain functional activity to protect against acute Zika virus infection

**DOI:** 10.1038/s41598-026-35079-3

**Published:** 2026-01-05

**Authors:** Jaehui Kim, Wooseong Lee, Do Yeon Kim, Keun Bon Ku, Young-Chan Kwon, Kyun-Do Kim, Chonsaeng Kim, Dae-Gyun Ahn, Seong-Jun Kim, Sungjun Park

**Affiliations:** 1 Center for Infectious Disease Vaccine and Diagnosis Innovation (CEVI), Korea Research Institute of Chemical Technology, Daejeon, 34114 Republic of Korea; 2 Untreatable Infectious Disease Institute, Kyungpook National University, Daegu, 41944 Republic of Korea; 3 Department of Microbiology, School of Medicine, Kyungpook National University, Daegu, 41944 Republic of Korea

**Keywords:** Zika virus (ZIKV), Immune cell infiltration, CD8^+^ T cells, T cell trafficking, Neuroinflammation, Cytotoxicity, *Ifnar1*^−^/^−^ mouse model, Acute infection, Immunology, Microbiology, Neuroscience

## Abstract

Zika virus (ZIKV) infection can cause severe neurological complications, yet the role of CD8^+^ T cells in controlling viral pathogenesis in the brain remains unclear. Using *Ifnar1*^−^/^−^ mice, which lack type I interferon signaling, we demonstrate that ZIKV infection triggers significant infiltration of CD8^+^ T cells into the brain, accompanied by neurological defects. ZIKV-experienced CD8^+^ T cells exhibit enhanced cytotoxic potential, and adoptive transfer of these cells improves survival. In contrast, blocking their infiltration exacerbates brain inflammatory and injury-associated signatures, highlighting their protective contribution. Furthermore, PD-1 blockade worsens ZIKV pathology, suggesting that PD-1 expression reflects an activated rather than exhausted state. These findings underscore an important role of infiltrating CD8^+^ T cells in reducing ZIKV-induced CNS inflammation and suggest that modulating their response could serve as a potential therapeutic strategy for ZIKV-associated neurological disease.

**Supplementary Information:**

The online version contains supplementary material available at 10.1038/s41598-026-35079-3.

## Introduction

ZIKV is a positive-sense RNA *flavivirus* that gained global attention during the 2015–2016 outbreak in Brazil, where approximately 2,000 cases of microcephaly were reported out of an estimated 200,000 ZIKV infections^1^. First identified in sentinel monkeys in Uganda in 1947^2^, ZIKV was later detected in humans in Nigeria in 1954^3^, and outbreaks have since occurred worldwide. ZIKV is primarily transmitted through the bite of *Aedes* spp. mosquitoes but can also spread via sexual contact, vertical transmission from mother to fetus, and blood transfusions^4^. Viral RNA and infectious ZIKV particles have been detected in the brains of fetuses with central nervous system (CNS) abnormalities, as well as in adult patients, where infection is associated with neurological complications such as Guillain-Barré syndrome^5–7^.

The blood-brain barrier (BBB) regulates the selective exchange of macromolecules and immune cells between the circulation and the CNS^8^. ZIKV has been shown to cross the BBB, leading to neuronal infection and damage^9,10^. Increasing evidence suggests that ZIKV employs a “Trojan horse” mechanism, wherein infected leukocytes infiltrate the CNS, facilitating viral dissemination and potentially serving as a reservoir^11^. Similar to West Nile virus (WNV), which infects monocytes and contributes to viral spread in the brain, leading to encephalitis and meningitis^12^, both ZIKV and dengue virus (DENV) also target monocytes^13,14^. Once inside the brain, ZIKV preferentially infects neural progenitor cells, leading to apoptosis, neuroinflammation, and developmental abnormalities^15^. Despite the severe consequences of ZIKV infection, no approved vaccines or antiviral therapies are currently available.

While neutralizing antibodies remain a major focus of vaccine development, cellular immunity, particularly CD8^+^ T cells, plays a crucial role in antiviral defense against *flaviviruses*^16,17^. In WNV and DENV infections, virus-specific CD8^+^ T cells contribute to viral clearance and protective immunity, with studies highlighting their importance in controlling viral replication in the CNS^18–21^. In ZIKV infection, CD8^+^ T cell responses have been identified^22^, and ZIKV-specific CD8^+^ T cells have been shown to recognize conserved viral epitopes and expand upon infection^23,24^; however, their functional role, particularly in providing effective protection in the CNS, remains largely unexplored. Given the similarities between ZIKV and other *flaviviruses*, as well as shared vaccine strategies^16,17,25^, further investigation into the contribution of CD8^+^ T cells in ZIKV pathogenesis is critical.

In this study, we investigate the contribution of CD8^+^ T cells to ZIKV infection using the *Ifnar1*^−^/^−^ mouse model. We show that ZIKV infection induces robust infiltration of CD8^+^ T cells into the brain, with ZIKV-experienced CD8^+^ T cells exhibiting enhanced cytotoxic potential. Furthermore, we found that these cells play a protective contribution in limiting ZIKV pathogenesis, as their depletion leads to worsened disease outcomes. These findings suggest a key role of CD8^+^ T cells in antiviral immunity against ZIKV and provide insights for future T cell-based therapeutic strategies.

## Results

### ZIKV infection induces brain inflammation-related responses and triggers anti-viral immune responses in *Ifnar1*^−^/^−^ mice

**Fig. 1:**
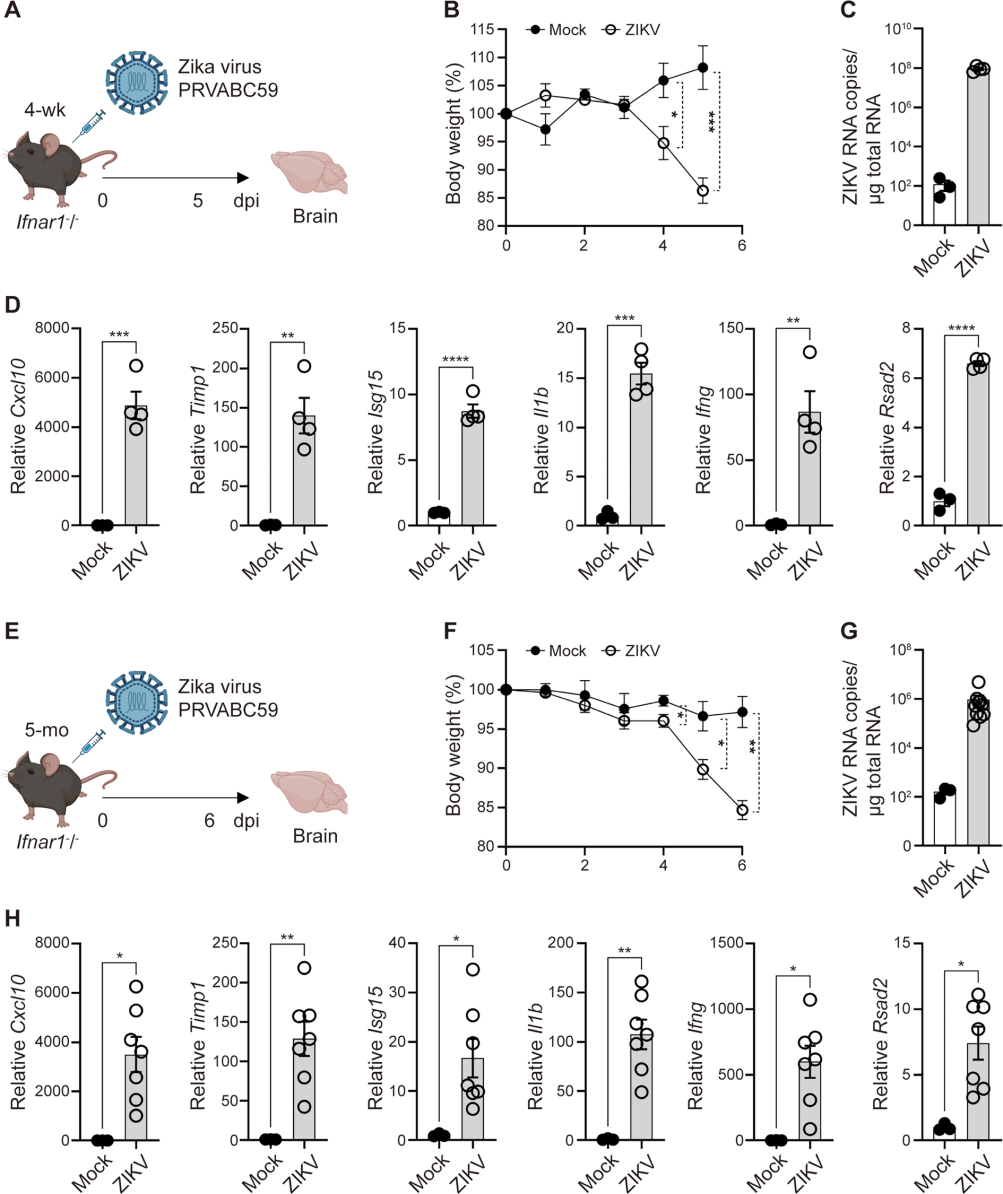
ZIKV infection is associated with brain pathology-related responses in both juvenile and mature adult *Ifnar1*^−^/^−^ mice. (**A**–**D**) Four-week-old *Ifnar1*^−^/^−^ mice were infected intraperitoneally (i.p.) with the ZIKV PRVABC59 strain. (**A**) Schematic representation of the experimental strategy for ZIKV infection in juvenile mice. (**B**) Daily body weight changes during ZIKV infection. Error bars indicate SEM. (**C**) qRT-PCR analysis of ZIKV RNA levels in the brain at 5 days post-infection. (**D**) qRT-PCR analysis of neuronal injury-related and antiviral immune responses in the brain at 5 days post-infection. Data are representative of more than two independent experiments. (**E**–**H**) Five-month-old *Ifnar1*^−^/^−^ mice were infected with the ZIKV PRVABC59 strain. (**E**) Schematic representation of the experimental strategy for ZIKV infection in mature adult mice. (**F**) Daily body weight changes during ZIKV infection. Error bars indicate SEM. (**G**) qRT-PCR analysis of ZIKV RNA levels in the brain at 6 days post-infection. All juvenile and adult brain samples were analyzed on the same qRT-PCR plate using a shared standard curve. Mock control values (~ 100 copies) are displayed to provide a visual baseline for viral RNA levels. (**H**) qRT-PCR analysis of genes associated with neuronal injury and antiviral immune responses in the brain at 6 days post-infection. Data are representative of two independent experiments. All data are presented as mean ± SEM. *P < 0.05; **P < 0.01; ***P < 0.005; ****P < 0.001 (two-tailed two-sample unequal variance Student *t* test).

Immune-competent adult mice are largely resistant to ZIKV infection because ZIKV cannot effectively suppress murine STAT2-dependent type I IFN responses. Although ZIKV NS5 degrades human STAT2 for immune evasion, this mechanism is inefficient in mice^26–30^. To overcome this limitation, we used *Ifnar1*^−^/^−^ mice, which lack type I IFN receptors and are highly susceptible to ZIKV infection^28^. Four-week-old *Ifnar1*^−^/^−^ mice were infected intraperitoneally (i.p.) with the ZIKV PRVABC59 strain, a clinical isolate obtained in 2015 from a patient in Puerto Rico (Fig. 1A)^31^. Body weight was monitored daily, and ZIKV-infected mice exhibited weight loss beginning at 4 days post-infection (Fig. 1B). ZIKV RNA was detected in the brains of infected mice 5 days post-infection (Fig. 1C). Given that ZIKV can infect the CNS and induce neurological abnormalities across developmental stages—from fetal development to adulthood—highlighting its neurotropic nature^32–34^, we next examined whether ZIKV infection induces neurological defects in our model.

CXCL10 is a well-established biomarker of acute ZIKV infection and is also linked to neuronal damage^35,36^, while TIMP1 contributes to neuroprotection by mitigating blood-brain barrier (BBB) disruption^37^. ISG15, a key restrictor of ZIKV replication, may likewise serve as a biomarker for BBB disruption during brain injury^38^; although typically induced by type I interferons, its expression can also be upregulated through type III IFN signaling or inflammation-related pathways, even without type I IFN receptor signaling^39,40^. The pro-inflammatory cytokine IL-1β is also recognized as a major contributor to brain injury^41^. Both our experimental data and previously published publicly accessible transcriptome datasets demonstrated that these brain inflammatory and injury-associated genes are markedly upregulated in the brains of ZIKV-infected mice compared with mock controls (Fig. 1D and Supplementary Fig. 1), indicating that ZIKV infection induces CNS inflammation. Notably, a previous study using a comparable *Ifnar1*^−^/^−^ ZIKV infection model reported typical virus-induced neuropathology, including meningoencephalitis and perivascular cuffing^42^, suggesting that similar histological changes are likely to accompany the inflammatory signatures observed in our system. In addition, *Ifng* and the interferon-inducible *Rsad2* gene, both of which enhance antiviral immune responses^43–45^, were also significantly increased in the brains of ZIKV-infected mice (Fig. 1D and Supplementary Fig. 1). We further examined whether these pathogenesis phenotypes were consistent in mature adult mice (Fig. 1E). Five-month-old *Ifnar1*^−^/^−^ mice infected with ZIKV showed similar outcomes: weight loss (Fig. 1F), ZIKV RNA detection in the brain (Fig. 1G), and increased expression of genes associated with CNS inflammation and antiviral immune responses (Fig. 1H). Although ZIKV RNA was detected in both juvenile and adult mice, its levels were higher in juveniles (Fig. 1C and G). In addition, body weight loss began earlier in juvenile mice, reaching approximately 85% of the initial weight by 5 days post-infection (dpi), whereas this occurred at 6 dpi in mature adult mice (Fig. 1B and F). These findings suggest that juvenile mice are more susceptible to ZIKV infection than mature adults when challenged with the same viral dose. Furthermore, C57BL/6 mice treated with an α-IFNAR-1 blocking antibody exhibited ZIKV pathogenesis and CNS inflammation similar to that observed in ZIKV-infected *Ifnar1*^−^/^−^ mice (Supplementary Fig. 2). Collectively, these findings indicate that ZIKV infection exacerbates brain neuroinflammation while simultaneously triggering antiviral immune responses.

### ZIKV infection promotes the recruitment of CD8^+^ T cells to the brain in *Ifnar1*^−^/^−^ mice

**Fig. 2:**
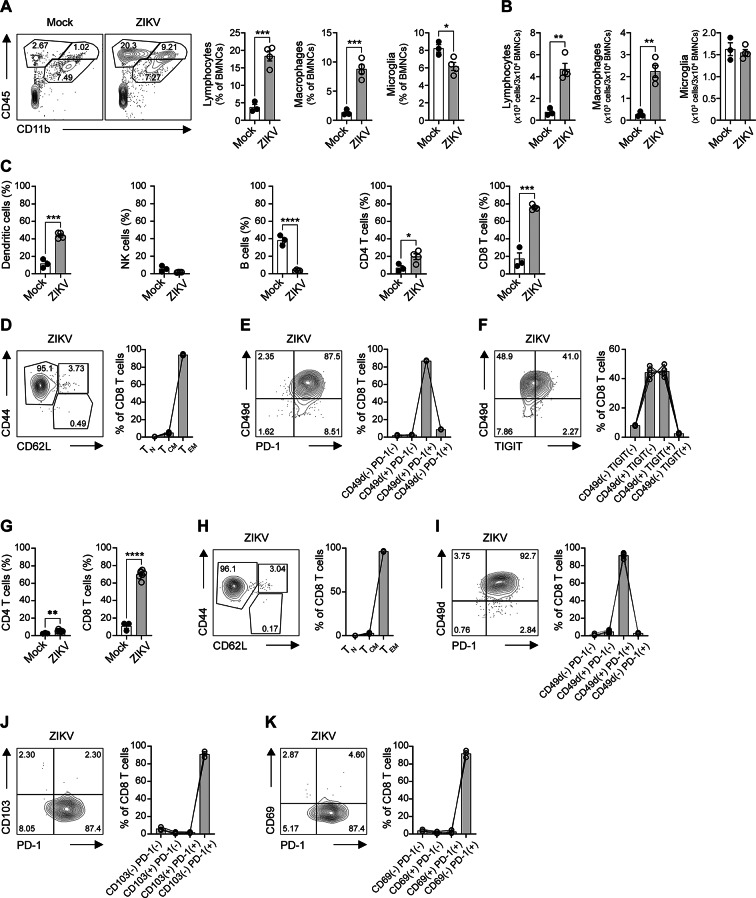
ZIKV infection enhances the expansion of brain-infiltrating CD8^+^ T cells in *Ifnar1*^−^/^−^ mice. (**A**–**F**) Four-week-old *Ifnar1*^−^/^−^ mice were infected with ZIKV. Five days post-infection, brains were harvested, and immune cells were analyzed by flow cytometry (FACS). (**A**) Representative FACS plots of brain cell suspensions from mock and ZIKV-infected mice (left) and quantification of the indicated populations (right). Lymphocytes were defined as CD45^hi^ CD11b^−^, macrophages (MΦ) as CD45^hi^ CD11b^+^, and microglia as CD45^int^ CD11b^+^. Data are shown as % of brain mononuclear cells (BMNCs). (**B**) Normalized counts for each population (x10^3^ cells per 3 × 10^4^ BMNCs). Events were gated on singlets (doublets excluded by FSC-H/FSC-A and SSC-H/SSC-A). (**C**) Percentages of dendritic cells (DCs), NK cells, B cells, CD4^+^ T cells, and CD8^+^ T cells within the lymphocyte gate at 5 days post infection. (**D**) Representative FACS plots (left) and bar graphs (right) depicting the frequencies of naïve CD8^+^ T cells (T_N_), central memory CD8^+^ T cells (T_CM_), and effector memory CD8^+^ T cells (T_EM_) in the brain of ZIKV-infected mice. (**E**) CD8^+^ T cells were analyzed based on the expression of CD49d and PD-1. Representative FACS plots (left) and bar graphs (right) are shown. (**F**) CD8^+^ T cells were analyzed based on the expression of CD49d and TIGIT. Data are representative of more than two independent experiments. (**G**–**K**) Five-month-old *Ifnar1*^−^/^−^ mice were infected with ZIKV. Six days post-infection, immune cells were analyzed in the brain by FACS. (**G**) Frequency of CD4^+^ or CD8^+^ T cells among brain-infiltrating lymphocytes (CD45^hi^ CD11b^−^). (**H**) Representative FACS plots (left) and bar graphs (right) depicting the frequencies of T_N_, T_CM_, and T_EM_ in the brain of ZIKV-infected mice. (**I**) CD8^+^ T cells were analyzed based on the expression of CD49d and PD-1. Representative FACS plots (left) and bar graphs (right) are shown. (**J**) CD8 T cells were analyzed based on the expression of CD103 and PD-1. (**K**) CD8^+^ T cells were analyzed based on the expression of CD69 and PD-1. Data are representative of two independent experiments. All data are presented as mean ± SEM. *P < 0.05; **P < 0.01; ***P < 0.005; ****P < 0.001 (two-tailed two-sample unequal variance Student *t* test).

Previous RNA-seq data showed that monocytes, macrophages, CD4^+^ T cells, and CD8^+^ T cells constitute the majority of immune cells in ZIKV-infected mice^46^. In other *flavivirus* infections, such as WNV, CXCL10 produced by infected neurons recruits CXCR3^+^ CD8^+^ T cells into the CNS^47,48^. A similar induction of CXCL10 has also been reported in ZIKV-infected hippocampal tissue^49^. Given the markedly elevated *Cxcl10* expression in ZIKV-infected brains (Fig. 1D H), we hypothesized that a similar CXCL10–CXCR3 axis mediates CD8^+^ T cell infiltration. Consistent with this, CD8^+^ T cells are recruited to the brain and spinal cord following ZIKV invasion^50–52^. To further characterize the immune landscape in the brain during the juvenile stage of infection, we analyzed brain immune cells by flow cytometry (FACS) (Supplementary Fig. 3). Brain mononuclear cells (BMNCs) were categorized into three subsets based on CD45 and CD11b expression: lymphocytes, macrophages (MΦ), and microglia (Supplementary Fig. 3). Lymphocytes and macrophages were markedly expanded in the brains of 4-week-old ZIKV-infected mice at 5 dpi relative to the mock group (Fig. 2A). By contrast, although the proportion of microglia was reduced in ZIKV-infected brains, their normalized counts were comparable between the mock and ZIKV-infected groups (Fig. 2B), consistent with previous observations^53,54^. Because activated microglia can upregulate CD45 and enter the CD45^hi^ gate^55^, we acknowledge that our gating strategy may miss a subset of activated microglia. Within the lymphocyte population, dendritic cells (DCs), CD4^+^ T cells, and CD8^+^ T cells were significantly increased in ZIKV-infected juvenile mice (Fig. 2C). Publicly available transcriptome datasets further corroborated these findings, showing enhanced antigen presentation, increased CD8^+^ T cell abundance, and elevated TCR signaling in infected brains (Supplementary Fig. 1), consistent with CD8^+^ T cell infiltration during ZIKV infection.

Given that CD8^+^ T cells were the predominant lymphocyte population in the brain at 5 days after ZIKV infection (Fig. 2C), we further analyzed them based on CD44 and CD62L expression. Most CD8^+^ T cells in ZIKV-infected brains displayed a CD44^+^ CD62L^–^ effector memory (T_EM_) phenotype (Fig. 2D). CD49d is a marker distinguishing antigen-experienced (CD49d^+^) from antigen-inexperienced (CD49d^–^) T cells, being upregulated upon TCR engagement and linked to effector cytokine production such as IFN-γ and TNF-α^56,57^. Conversely, CD49d^–^ T cells are typically considered virtual memory cells, supporting CD49d as a reliable marker of true antigen experience^58–60^. Importantly, in ZIKV-infected mice, antigen-experienced CD49d^+^ CD8^+^ T cells represented the majority of brain-infiltrating CD8^+^ T cells and expressed PD-1 (Fig. 2E), a marker typically induced during acute viral activation, but also detectable in early exhaustion states^61^. Additionally, approximately 50% of CD49d^+^ CD8^+^ T cells co-expressed TIGIT, another activation marker, in the brains of 4-week-old ZIKV-infected mice (Fig. 2F). Similar to juvenile mice, 5-month-old adults also exhibited an increased frequency of brain-infiltrating CD8^+^ T cells (Fig. 2G), the majority of which were T_EM_ cells (Fig. 2H) and antigen-experienced, activated CD49d^+^ PD-1^+^ T cells (Fig. 2I). These CD8^+^ T cells expressed very low levels of CD103 (Fig. 2J) and CD69 (Fig. 2K), well-recognized markers of tissue-resident memory T (T_RM_) cells in the brain^62^, indicating they were recent infiltrates rather than resident cells. Although type I IFN signaling can differentially affect intestinal T_RM_ subsets^63^, its impact on brain T_RM_ differentiation during ZIKV infection remains unclear. Thus, CD69 and CD103 patterns in our model should be interpreted with this potential limitation in mind. In addition to CD8^+^ T cells, CD4^+^ T cells were also recruited to the brain following ZIKV infection, although they accounted for less than 10% of total infiltrating lymphocytes in mature adult mice (Fig. 2G). Most brain-infiltrating CD4^+^ T cells displayed an effector/memory (T_EFF_) phenotype, and 40–70% of them were antigen-experienced (CD49d^+^) in both juvenile and adult mice (Supplementary Fig. 4). Nonetheless, CD8^+^ T cells remained the predominant lymphocyte population at both ages (Fig. 2C and G), and therefore our subsequent analyses primarily focused on CD8^+^ T cell responses during acute ZIKV infection. Consistent with these findings, analysis of lymphocyte lineage marker transcripts showed that CD8-associated genes were the most strongly upregulated in infected brains (Supplementary Fig. 2E and Supplementary Fig. 5). Human Protein Atlas data show that *PDCD1* is largely restricted to T cells and rarely expressed in normal brain tissue^64^, consistent with the immune-privileged nature of the brain, which typically limits T cell infiltration. However, in ZIKV-infected mice, *Pdcd1* expression was highly upregulated in the brain, and nearly all brain-infiltrating CD8^+^ T cells expressed PD-1 (Fig. 2E and I, Supplementary Fig. 1, and Supplementary Fig. 6). These findings suggest that PD-1-expressing CD8^+^ T cells are actively recruited to the brain during acute ZIKV infection.

### ZIKV-experienced CD8 T cells exhibit enhanced cytotoxicity

**Fig. 3:**
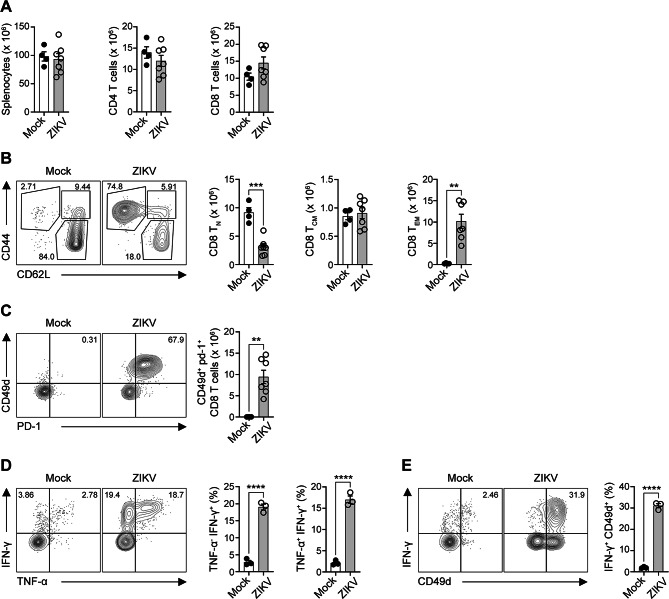
ZIKV-experienced CD8^+^ T cells exhibit increased cytotoxic potential through enhanced cytokine production. (**A**–**E**) Five-month-old *Ifnar1*^−^/^−^ mice were infected with ZIKV, and spleens were harvested for immune cell analysis at 6 days post-infection. (**A**) Absolute numbers of splenocytes, CD4^+^ T cells, and CD8^+^ T cells in the spleens of *Ifnar1*^−^/^−^ mice. (**B**) Representative FACS plots (left) and bar graphs (right) depicting the frequencies of T_N_, T_CM_, and T_EM_ in the spleens of mock and ZIKV-infected mice. (**C**) Representative FACS plots (left) and bar graphs (right) depicting the frequencies of CD49d^+^ PD-1^+^ CD8^+^ T cells in the spleens of *Ifnar1*^−^/^−^ mice. (**D**,**E**) Splenocytes from mock and ZIKV-infected mice were stimulated with PMA and ionomycin in the presence of GolgiStop (containing monensin) and GolgiPlug (containing brefeldin A) for 5 h. (**D**) Flow cytometry analysis of IFN-γ and TNF-α production by CD8^+^ T cells. (**E**) Frequency of IFN-γ-expressing CD49d^+^ CD8^+^ T cells in the spleen. Data are representative of two independent experiments. All data are presented as mean ± SEM. **P < 0.01; ***P < 0.005; ****P < 0.001 (two-tailed two-sample unequal variance Student *t* test).

To elucidate the characteristics of ZIKV-experienced CD8⁺ T cells, we analyzed immune cells in the spleen, as the brain lacks lymphatic vessels and immune entry mainly occurs across the BBB^65^. The total numbers of splenocytes, CD4⁺ T cells, and CD8⁺ T cells were comparable between the mock and ZIKV-infected groups (Fig. 3A). However, CD44^+^ CD62L^−^ T_EM_ cells were significantly upregulated at the expense of CD44^−^ CD62L^+^ naïve CD8^+^ T cells in the spleens of ZIKV-infected mice compared to the mock group (Fig. 3B). Notably, CD49d^+^ CD8^+^ T cells were nearly absent in the mock group but accounted for more than 65% of CD8^+^ T cells in ZIKV-infected mice, suggesting these antigen-experienced cells could serve as a source of brain-infiltrating cells (Fig. 3C).

To assess their functional properties, we analyzed cytokine production by FACS. ZIKV infection led to a significant increase in IFN-γ and TNF-α production by CD8^+^ T cells (Fig. 3D), with IFN-γ being predominantly produced by CD49d^+^ CD8^+^ T cells (Fig. 3E). ZIKV-stimulated IFN-γ–producing CD8^+^ T cells are known to exhibit enhanced cytotoxic and activation properties, underscoring their effector potential (Supplementary Fig. 7). Given that both cytokines are hallmarks of cytotoxic effector function, these results indicate that ZIKV-experienced CD49d^+^ CD8^+^ T cells possess potent cytotoxic capacity. This population may subsequently migrate to the brain, where most CD8^+^ T cells express the CD49d marker (Fig. 2E and I).

In addition to CD8^+^ T cells, ZIKV infection also expanded splenic CD4^+^ T_EFF_ cells and increased the frequency of ZIKV-experienced CD4^+^ T cells (Supplementary Fig. 8A and 8B). These CD4^+^ T cells produced IFN-γ following ZIKV infection (Supplementary Fig. 8C), suggesting that they might also undergo a degree of functional activation; however, the magnitude of this cytokine response was substantially lower than that observed in CD8^+^ T cells (Fig. 3E). The functional significance of CD4^+^ T cell activation during acute ZIKV infection remains to be clarified.

### ZIKV-experienced CD8 T cells mitigate ZIKV pathogenesis in *Ifnar1*^−^/^−^ mice

**Fig. 4:**
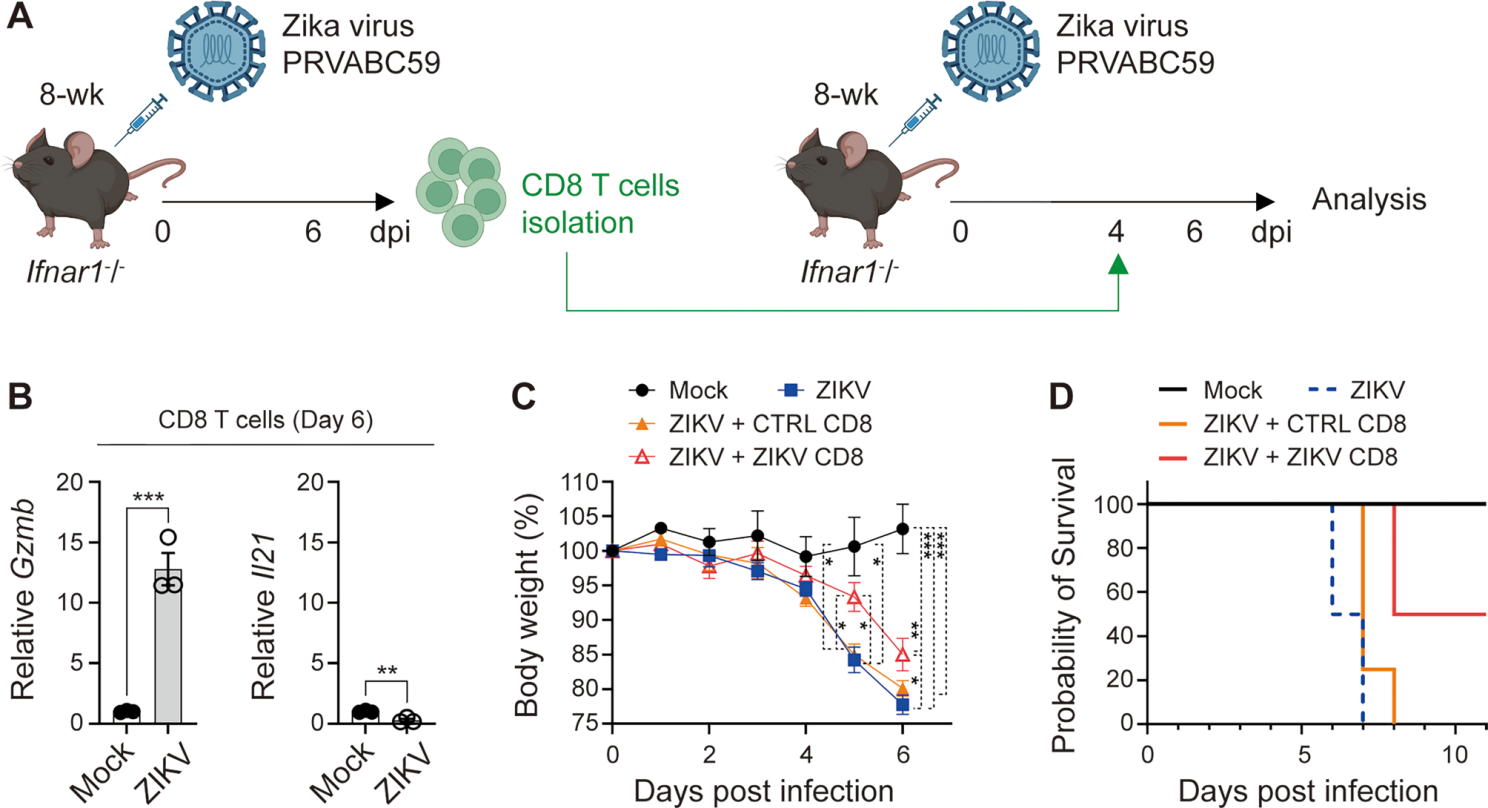
ZIKV-experienced CD8^+^ T cells mitigate body weight loss and improve survival outcomes. (**A**–**D**) Eight-week-old *Ifnar1*^−^/^−^ mice were infected with ZIKV, and at 6 days post-infection, splenic CD8⁺ T cells were isolated and intravenously transferred into *Ifnar1*^−^/^−^ mice that had been infected with the same virus 4 days earlier. (**A**) Schematic illustration of the experimental mouse model. (**B**) qRT-PCR analysis of *Gzmb* and *Il21* expression in isolated CD8^+^ T cells at 6 days post-infection. (**C**) Daily body weight changes during ZIKV infection. Error bars indicate SEM. (**D**) Survival rates of the mock group, ZIKV-infected group, ZIKV-infected group receiving control CD8^+^ T cells (CTRL CD8), and ZIKV-infected group receiving ZIKV-experienced CD8^+^ T cells (ZIKV CD8). The survival curves were significantly different among the four groups (log-rank [Mantel–Cox] test: χ² = 16.80, df = 3, *p* = 0.0008). Data are representative of two independent experiments. All data are presented as mean ± SEM. *P < 0.05; **P < 0.01; ***P < 0.005 (two-tailed two-sample unequal variance Student *t* test).

Since ZIKV-experienced CD8^+^ T cells exhibit cytotoxic activity, we sought to determine whether they play a protective role in vivo during ZIKV infection. We used 8-week-old donor mice for the adoptive transfer experiment because young-adult mice exhibit robust adaptive immune competence before age-related immunosenescence begins^66^. Using donors at this stage ensures optimal T-cell functionality for adoptive transfer. To test this, *Ifnar1*^−^/^−^ mice were infected with ZIKV, and at 6 days post-infection, CD8^+^ T cells were isolated and transferred into recipient *Ifnar1*^−^/^−^ mice that had been infected with the same virus 4 days earlier (Fig. 4A). Isolated ZIKV-infected CD8^+^ T cells (ZIKV CD8) exhibited high cytotoxicity, as indicated by a significant upregulation of *Gzmb* (Fig. 4B), a key effector molecule involved in the elimination of infected cells^67^, compared to CD8^+^ T cells from uninfected control mice (CTRL CD8). In contrast, the expression of *Il21*, a cytokine typically associated with helper-like functions^68^, was reduced in ZIKV CD8 T cells relative to CTRL CD8 T cells, suggesting a functional skewing toward a cytotoxic rather than helper phenotype. Both ZIKV-infected mice and those receiving CTRL CD8 T cells displayed significant weight loss relative to mock controls. By contrast, mice administered ZIKV CD8 T cells post infection showed markedly reduced weight loss (Fig. 4C). Additionally, ZIKV-infected mice that received ZIKV CD8 T cells demonstrated an improved survival rate compared to those administered CTRL CD8 T cells (Fig. 4D). An independent study likewise showed that CD8^+^ T cell depletion increases brain viral burdens and mortality during primary ZIKV infection^69^, supporting an important protective contribution of CD8^+^ T cells in vivo. Notably, adoptive transfer studies also show that ZIKV-immune CD8^+^ T cells lower viral loads in multiple tissues—including the brain, kidney, and blood—although they do not fully clear infection^23^. This implies that transferred CD8^+^ T cells enhance systemic viral control, while the exact site of their protective activity remains to be clarified. Together, these results suggest that ZIKV-experienced CD8^+^ T cells confer protective immunity when adoptively transferred, effectively mitigating disease severity and improving survival outcomes.

### Reduced infiltration of CD8^+^ T cells aggravates ZIKV infection

**Fig. 5:**
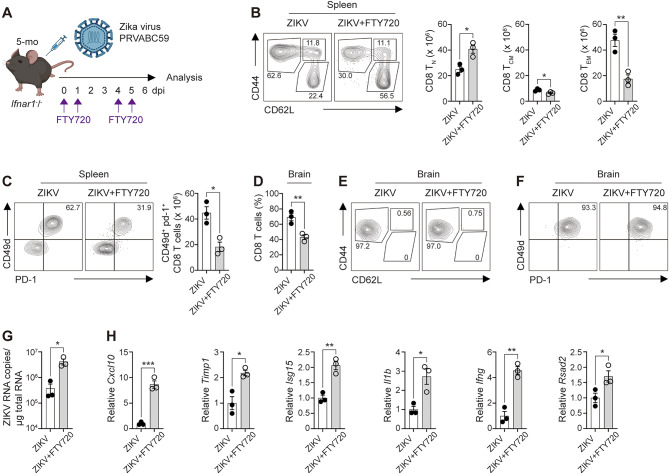
Reduced brain-infiltrating CD8^+^ T cells are associated with exacerbated ZIKV-induced brain pathology. (**A**–**H**) Five-month-old *Ifnar1*^−^/^−^ mice were infected with the ZIKV PRVABC59 strain. To block T cell egress into circulation, FTY720 was administered on days 0, 1, 4, and 5 post-infection. At six days post-infection, spleen and brain tissues were harvested for analysis. (**A**) Schematic illustration of the experimental mouse model. (**B**) Representative FACS plots (left) and bar graphs (right) showing the absolute numbers of T_N_, T_CM_, and T_EM_ CD8^+^ T cells in the spleen of ZIKV-infected mice with or without FTY720 treatment. (**C**) Representative FACS plots (left) and bar graphs (right) depicting the absolute numbers of CD49d^+^ PD-1^+^ CD8^+^ T cells in the spleen of ZIKV-infected mice. (**D**) Frequency of CD8^+^ T cells within the brain lymphocyte gate (CD45^hi^ CD11b^−^). (**E**) Representative FACS plots showing the distribution of T_N_, T_CM_, and T_EM_ subsets in the brain of ZIKV-infected mice. (**F**) Representative FACS plots of CD49d^+^ PD-1^+^ CD8^+^ T cells in the brain. (**G**) qRT-PCR analysis of ZIKV RNA levels in the brain at six days post-infection. (**H**) qRT-PCR analysis of genes associated with neuronal injury and antiviral immune responses in the brain at six days post-infection. Data are representative of two independent experiments. All data are presented as mean ± SEM. *P < 0.05; **P < 0.01; ***P < 0.005 (two-tailed two-sample unequal variance Student *t* test).

Based on our findings, we hypothesized that cytotoxic CD8^+^ T cells infiltrate the brain, where they may contribute to protection against neuroinflammatory and injury-associated processes. To test this, we administered fingolimod (FTY720), a sphingosine 1-phosphate receptor-1 (S1PR1) agonist that prevents immune cell egress into inflamed tissues^70^, during ZIKV infection and analyzed T cell responses and CNS inflammatory changes (Fig. 5A). We used 5-month-old mice for this experiment because this age represents a fully mature adult with a stable immune system, enabling a more physiologically reliable assessment of FTY720-mediated T-cell egress blockade. Six days post-infection, mice treated with FTY720 exhibited a reduction in splenic CD44^+^ CD62L^−^ T_EM_ cells (Fig. 5B) and splenic ZIKV-experienced CD49d^+^ CD8^+^ T cells (Fig. 5C) compared to untreated mice, indicating that FTY720 effectively blocked T cell egress into circulation. In addition to its effects on CD8^+^ T cells, FTY720 treatment also reduced the frequencies of splenic T_EFF_ CD4^+^ T cells and ZIKV-experienced CD4^+^ T cells (Supplementary Fig. 9A and 9B). Whereas the frequency of brain-infiltrating CD8^+^ T cells was significantly decreased in FTY720-treated mice, the frequency of brain-infiltrating CD4^+^ T cells remained unchanged (Fig. 5D and Supplementary Fig. 9C), suggesting that CD8^+^ T cells may represent the subset most sensitive to S1PR1-dependent inhibition of lymphocyte egress into the brain. Those CD8^+^ T cells that did infiltrate still predominantly displayed T_EM_ and ZIKV-experienced phenotypes (Fig. 5E and F). Notably, ZIKV RNA levels increased approximately 10-fold in the brains of FTY720-treated mice compared to untreated controls (Fig. 5G). Furthermore, genes associated with CNS inflammation and antiviral immune responses were upregulated in the brains of FTY720-treated mice following ZIKV infection (Fig. 5H). These findings suggest that limiting CD8^+^ T cell infiltration exacerbates ZIKV-induced neuroinflammation and that enhancing CD8^+^ T cell access to the CNS may represent a potential strategy for improving vaccine efficacy.

### PD-1 blockade in ZIKV-infected *Ifnar1*^−^/^−^ mice exacerbates ZIKV pathogenesis

**Fig. 6:**
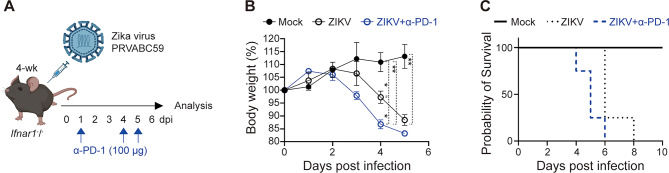
PD-1 blockade aggravates ZIKV pathogenesis in *Ifnar1*^−^/^−^ mice. (**A**–**C**) Four-week-old *Ifnar1*^−^/^−^ mice were infected with the ZIKV PRVABC59 strain. To assess the effects of PD-1 blockade, α-PD-1 antibody was administered on days 1, 4, and 5 post-infection. (**A**) Schematic illustration of the experimental mouse model. (**B**) Daily body weight changes during ZIKV infection. Error bars indicate SEM. (**C**) Survival rates of mock, ZIKV-infected, and ZIKV-infected mice treated with α-PD-1 antibody. The survival curves differed significantly among the three groups (log-rank [Mantel–Cox] test: χ² = 13.05, df = 2, *p* = 0.0015). Data are representative of two independent experiments. *P < 0.05; **P < 0.01 (two-tailed two-sample unequal variance Student *t* test).

Although PD-1 is a well-characterized inhibitory receptor typically associated with T cell exhaustion in chronic infections^71^, accumulating evidence indicates that PD-1^+^ CD8^+^ T cells can remain functional during acute viral infections and may contribute to protective immunity^72–74^. For example, PD-1^+^ CD8^+^ T cells in SARS-CoV-2 infection retained activation and effector capacity despite high PD-1 expression^74^. Similarly, CD8^+^ T cells from ZIKV-infected patients displayed enhanced cytotoxic and activation profiles alongside upregulation of inhibitory receptors (Supplementary Fig. 5). Consistently, our data show that ZIKV-experienced PD-1^+^ CD8^+^ T cells retain strong effector activity during acute infection (Fig. 3C and E). While this supports functional engagement rather than dysfunction, our data cannot fully exclude early exhaustion-like states. To assess their functional role, we administered an α-PD-1 blocking antibody to ZIKV-infected mice during the acute phase and monitored disease progression up to 6 dpi (Fig. 6A). Treated mice exhibited greater weight loss and significantly reduced survival compared with ZIKV-infected controls (Fig. 6B C), suggesting that PD-1 signaling contributes to protective regulation during acute infection. However, as our analysis centered on clinical outcomes, whether this worsening reflects reduced viral control or increased immunopathology remains unclear.

In contrast, a glioblastoma (GBM) tumor model study presents a distinct scenario for PD-1^+^ CD8^+^ T cells following ZIKV treatment^75^. In that setting, viral RNA became undetectable by 14 days post-treatment, yet CD8^+^ T cells remained elevated within the tumor microenvironment, suggesting that under prolonged, virus-free conditions these cells may undergo reduced effector activity or transition toward exhaustion. Unlike this chronic, virus-cleared context, our study focuses on the acute phase, where PD-1^+^ CD8^+^ T cells remain effector-competent and protective. Accordingly, PD-1 blockade during this phase interferes with their function and exacerbates disease (Fig. 6B C), underscoring the importance of infection timing and immune context in defining PD-1 function. Moreover, studies in persistent viral encephalitis have shown that PD-1 limits immunopathology and supports the development and maintenance of brain-resident memory CD8^+^ T cells^76,77^, reinforcing the concept that PD-1 can modulate T cell fate beyond exhaustion depending on tissue environment and infection stage.

## Discussion

Our findings demonstrate that ZIKV infection induces CNS inflammation and injury-associated molecular signatures and triggers an immune response, with ZIKV-experienced CD8^+^ T cell immunity playing a major role in combating acute viral infection in mice. These ZIKV-experienced and activated CD8^+^ T cells exhibit enhanced cytotoxicity, which likely contributes to reducing neuroinflammatory and injury-associated processes by infiltrating the brain. Importantly, limiting their access—either by preventing infiltration with FTY720 or blocking their function with an α-PD-1 antibody—exacerbated disease severity. Moreover, we show that PD-1–expressing CD8^+^ T cells retain robust effector activity during acute infection, although this does not fully exclude early exhaustion-like features.

In this study, we utilized different mouse strains and age groups to assess ZIKV pathogenesis. Our results indicate that younger mice exhibit more severe pathological phenotypes than adult mice when infected with the same viral titer, with approximately 50% survival at 6 days post-infection in young mice (data not shown). Based on this observation, immune profiling was conducted at 5 days post-infection. At this stage, CD8^+^ T cells already comprised ~ 50% of brain-infiltrating lymphocytes, a frequency that likely increases further by days 7–8, consistent with the peak of T cell expansion during viral infection^78^. Previous studies similarly reported robust CD8^+^ T cell responses in C57BL/6 mice, which correlated with mild disease manifestations^22^. In contrast, disruption of type I interferon (IFN-I) signaling using an α-IFNAR-1 antibody markedly increased susceptibility, paralleling the phenotype of *Ifnar1*^−^/^−^ mice. These findings highlight the cooperative roles of innate and adaptive immunity in controlling ZIKV pathogenesis.

Although our study emphasizes the dominant role of CD8^+^ T cells during acute ZIKV infection, our expanded analyses indicate that CD4^+^ T cells may also contribute to the immune response. CD4^+^ T cells were recruited to the brain but constituted < 10% of infiltrating lymphocytes and remained markedly fewer than CD8^+^ T cells. In both brain and spleen, they displayed effector/memory phenotypes, suggesting that they might undergo some degree of activation during infection. FTY720 reduced splenic CD4^+^ and CD8^+^ T cell populations, but only CD8^+^ T cells showed decreased frequencies in the brain, indicating that CD8^+^ T cells may be more sensitive to S1PR1-dependent egress blockade. Previous work has shown that CD4^+^ T cells are required primarily for the generation of ZIKV-specific humoral immunity rather than for supporting robust CD8^+^ T cell responses^79^, suggesting that their contribution may depend on infection route and immunological context. Thus, while CD4^+^ T cells might provide helper or inflammatory inputs, CD8^+^ T cells may represent an effector subset that contributes to antiviral control and modulates CNS inflammation in our model. Although FTY720 influences multiple circulating lymphocyte populations, CD8^+^ T cells appear to be the subset most affected in the infected brain, consistent with their dominant representation among infiltrating lymphocytes (Fig. 2C and G).

While our study primarily focuses on the role of CD8^+^ T cells during acute ZIKV infection, our model may also provide insights into memory CD8^+^ T cell responses during chronic infection. Indeed, memory CD8 T cells are generated during ZIKV infection, and adoptive transfer studies have shown that these cells are sufficient to confer protection against lethal ZIKV challenge^22,69^. It has been reported that brain-infiltrating CD8^+^ T cells can differentiate into CD69^+^ CD103^+^ T_RM_ cells in various disease settings^62,80–82^. Consistently, recent studies demonstrated that peripherally activated CD8^+^ T cells can give rise to brain-resident CD69^+^ CD103^+^ T_RM_ cells even without direct CNS infection, highlighting the potential for T_RM_ generation following peripheral viral challenge^83^. In our model, where i.p. ZIKV infection led to substantial brain infiltration by CD8^+^ T cells, a similar pathway of peripheral activation followed by T_RM_ differentiation is plausible. Furthermore, repetitive peripheral antigen stimulation has been shown to still allow the formation of protective brain-resident T_RM_ cells, though with altered phenotype and localization compared to those generated by a single exposure^84^. Elucidating the fate of these ZIKV-induced CD8^+^ T cells—including their maintenance, differentiation into memory subsets, and functional capacity upon secondary challenge—will be critical for understanding long-term immunity and for designing vaccines that promote durable CD8^+^ T cell memory.

Type I IFN deficiency markedly enhances ZIKV replication, alters viral tropism, and facilitates CNS invasion, as demonstrated in *Ifnar1*^−^/^−^ models^28^. Because IFN-I acts directly on CD8^+^ T cells to support their clonal expansion, survival, effector differentiation, and memory formation, the magnitude and phenotype of CD8^+^ T cell responses in IFNAR-deficient mice may differ substantially from those in immunocompetent hosts^85^. Moreover, IFN-I is required to protect antiviral CD8^+^ T cells from NK-cell–mediated deletion, raising the possibility that CD8^+^ T cell priming or persistence may be altered independently of viral neurotropism^86^. Recent work further shows that transient IFNAR blockade can shift CD8^+^ T cells toward stem-like memory fates, underscoring the broad influence of IFN-I on CD8^+^ T cell differentiation^87^. Together, these IFN-I–dependent, model-specific effects should be considered when interpreting CD8^+^ T-cell dynamics in our study.

Here, we primarily focused on the brain, a well-recognized immune-privileged organ. However, ZIKV is known to infect multiple tissues, including the testes, where viral persistence may lead to long-term reproductive consequences^88^. Indeed, CD8^+^ T cells infiltrate persistently infected testes and play a key role in viral clearance and tissue protection in mouse models^89^. Moreover, maternal-fetal transmission of ZIKV remains a critical concern, as infection during pregnancy can result in severe congenital abnormalities^90^. One study has shown that DENV/ZIKV cross-reactive CD8^+^ T cells can accumulate in the placenta, reduce viral burden, and improve fetal outcomes in a pregnancy model^91^. Thus, future studies should also investigate CD8^+^ T cell responses in other immune-privileged sites such as the testes and placenta, to better understand tissue-specific antiviral immunity in reproductive organs and inform vaccine strategies that enhance protection while minimizing immunopathology.

Our findings regarding PD-1 expression in CD8^+^ T cells further highlight the complexity of immune regulation in viral infections. While PD-1 is often associated with T cell exhaustion in chronic infections and cancer^75,92^, our data, together with previous studies^72–74^, indicate that PD-1 can also mark functionally active CD8^+^ T cells during acute infection. However, PD-1 expression alone cannot fully resolve whether these cells reflect a purely activated state or early exhaustion-like features, as our analyses primarily capture effector outputs rather than deeper transcriptional programs. Moreover, studies in models of persistent viral encephalitis have shown that PD-1 helps regulate neuroinflammation and facilitates the generation of brain-resident memory CD8^+^ T cells^76,77^. These observations underscore that PD-1 is not merely a marker of exhaustion but can influence T cell fate in a context-dependent manner, shaped by tissue microenvironment and infection stage (Supplementary Fig. 10). Although PD-1 blockade clearly worsened disease outcomes in our model, the present data do not determine whether this reflects impaired viral control or increased immunopathology, as additional virological and cellular analyses were not performed. Clarifying how PD-1 signaling shapes disease severity during acute ZIKV infection will be an important direction for future studies. A deeper understanding of how inhibitory receptors such as PD-1 modulate immunity in acute versus chronic infections will be crucial for developing immunotherapies that balance protective responses with the risk of immunopathology.

In conclusion, our study suggests that CD8^+^ T cells play a protective contribution during ZIKV infection in the brain and that PD-1–expressing CD8^+^ T cells retain strong effector function during acute infection. Future studies should also investigate CD8^+^ T cell responses in ZIKV-affected tissues beyond the brain and explore the long-term fate of ZIKV-experienced CD8^+^ T cells. Moreover, elucidating their roles in infections caused by related *flaviviruses*, such as DENV and yellow fever virus, will further expand our understanding. A comprehensive characterization of CD8^+^ T cell–mediated immunity against ZIKV will be essential for guiding the development of effective therapeutics and vaccines to combat ZIKV and other mosquito-borne viral diseases.

## Materials and methods

### Mice

*Ifnar1*^−^/^−^ mice were purchased from B&K Universal, Ltd. (Hull, UK) and bred and maintained in a BSL-2 animal facility at the Korea Research Institute of Chemical Technology (KRICT) under a standard 12-hour light/12-hour dark cycle. Mice were provided with standard rodent chow and water *ad libitum*. Three to seven male and female mice were randomly assigned to each experimental group. All animal procedures were approved by the KRICT Institutional Animal Care and Use Committee (protocol no. 8 A-M1) and were conducted in accordance with institutional guidelines and national regulations for animal research. The study is reported in accordance with the ARRIVE guidelines.

### Cell culture and virus preparation

Two ZIKV strains, PRVABC59 (VR-1843) and MR766 (VR-84) were purchased from the American Type Culture Collection (ATCC, Rockville, MD, USA). All ZIKV stocks were propagated on Vero cells (CCL-81, ATCC) cultivated in minimum essential medium (MEM, Gibco) supplemented with 10% fetal bovine serum (FBS, Gibco), 100 U/ml penicillin and 100 µg/ml streptomycin (Gibco) at 37 °C and 5% CO_2_ conditions. ZIKV stocks were concentrated with Amicon^®^ Ultra-15 Centrifugal Filter Units (Merck, Darmstadt, Germany).

### Virus infection

*Ifnar1*^−^/^−^ mice were infected intraperitoneally (i.p.) with 250 PFU of the ZIKV PRVABC59 strain in 100 µL under isoflurane inhalation anesthesia. The experimental endpoint was defined as either > 20% weight loss (in accordance with humane standards for laboratory animals at KRICT) or the development of hindlimb paralysis. Health status and body weight were monitored daily. At the indicated days post-infection, mice were euthanized, and brain and spleen tissues were collected for further analysis.

### Antibodies and flow cytometry

Single-cell suspensions of brain and spleen tissues were prepared and stained with monoclonal antibodies in fluorescence-activated cell sorting (FACS) buffer (phosphate-buffered saline (PBS) + 2.5% fetal bovine serum (FBS)). For brain tissue processing, brains were harvested and minced, followed by enzymatic digestion in RPMI-1640 medium supplemented with 10% FBS, Liberase (Roche, Cat. No. 5401127001), and DNase I (Roche, Cat. No. 11284932001) for 20 min at 37 °C with continuous shaking. The resulting cell suspensions were passed through a 70 μm cell strainer at least three times. Spleens were mechanically dissociated into single-cell suspensions and treated with red blood cell (RBC) lysis buffer to remove erythrocytes. Cells were then filtered through a 70 μm cell strainer before proceeding to FACS staining. The following monoclonal antibodies (purchased from BioLegend) were used for surface staining: anti-CD45 (30-F11, Cat. No. 103151); anti-CD11b (M1/70, Cat. No. 101211); anti-CD11c (N418, Cat. No. 117307); anti-B220 (RA3-6B2, Cat. No. 103206); anti-NK1.1 (S17016D, Cat. No. 156513); anti-CD4 (GK1.5, Cat. No. 100407); anti-CD8 (YTS156.7.7, Cat. No. 126609); anti-CD44 (IM7, Cat. No. 103032); anti-CD62L (MEL-14, Cat. No. 104412); anti-CD49d (R1-2, Cat. No. 103605); anti-PD-1 (29 F.1A12, Cat. No. 135231); anti-TIGIT (1G9, Cat. No. 142121); anti-CD69 (H1.2F3, Cat. No. 104506); anti-CD103 (2E7, Cat. No. 121422). For cytokine staining, total splenocytes were stimulated with PMA and ionomycin in the presence of GolgiStop (BD, Cat. No. 554724) and GolgiPlug (BD, Cat. No. 555029) for 5 h. Intracellular staining was performed using the Foxp3 Staining Buffer Set (eBioscience, Cat. No. 00-5523), followed by staining with anti-IFN-γ (XMG1.2, Cat. No. 505809) and anti-TNF-α (MP6-XT22, Cat. No. 506327). Fixable viability dyes (AF488 or BV786) were used to exclude dead cells. Live cells were first gated on FSC-A vs. SSC-A, and doublets were removed using FSC-H vs. FSC-A followed by SSC-H vs. SSC-A. Immune cell subsets were subsequently gated as shown in Supplementary Fig. 3. Stained cell samples were analyzed using a FACSymphony A3 (BD), and data were processed with FlowJo software (Tree Star).

### Isolation of CD8^+^ T cells and cell transfer into mice

Total CD8^+^ T cells were isolated from spleen single-cell suspensions to > 95% purity using a CD8^+^ T cell negative selection kit (Stem Cell Technologies). For adoptive transfer experiments, 2.5 × 10^5^ CD8^+^ T cells were isolated from *Ifnar1*^−^/^−^ mice 6 days post-infection with the ZIKV PRVABC59 strain. These cells were then intravenously (i.v.) transferred into recipient *Ifnar1*^−^/^−^ mice, which had been challenged with ZIKV 4 days prior. Control CD8^+^ T cells were isolated from uninfected, normal *Ifnar1*^−^/^−^ mice. In addition to adoptive transfer, isolated CD8^+^ T cells were subjected to qRT-PCR analysis to assess gene expression profiles.

### Depletion of immune cell subsets using FTY720 treatment

To prevent immune cell egress into circulation, *Ifnar1*^−^/^−^ mice were injected i.p. with FTY720 (1 mg/kg) at the indicated time points during ZIKV infection. On day 6 post-infection, spleen and brain tissues were harvested to assess T cell infiltration and neuronal damage.

#### In vivo PD-1 blockade treatment

To assess the effects of PD-1 blockade during acute ZIKV infection, *Ifnar1*^−^/^−^ mice were i.p. administered 100 µg of α-PD-1 antibody on days 1, 4, and 5 post-infection with the ZIKV PRVABC59 strain. Body weight and survival were monitored throughout the course of infection.

#### In vivo IFNAR1 blockade treatment

C57BL/6 mice were obtained from Orient Bio and housed in a BSL-2 animal facility at KRICT. To render mice susceptible to ZIKV infection, 250 µg of α-IFNAR1 antibody was administered i.p. on days − 1, 0, 1, 2, and 3 post-infection with 1000 PFU of the ZIKV MR766 strain. Body weight was monitored daily, and on day 7 post-infection, brain tissues were harvested for RNA analysis.

### Real-time RT-qPCR

Total RNA was extracted using RNeasy (Qiagen, Cat. No. 74106), then 0.2–1 µg of the total RNA was subjected to cDNA synthesis using a SuperScript Reverse Transcription System (Invitrogen, Cat. No. 18090200). Each gene expression level was normalized to *Gapdh* levels and presented as relative to mock group. Because multiple genes were evaluated in parallel, the multi-gene RT-qPCR panel was performed as an exploratory analysis. Accordingly, nominal *P* values are reported without adjustment for multiple comparisons and should be interpreted cautiously. The primer sequences used in the RT-qPCR analyses were as follows: *Gapdh* (forward: 5’ AGG TCG GTG TGA ACG GAT TTG 3’; reverse: 5’ TGT AGA CCA TGT AGT TGA GGT CA 3’), *Cxcl10* (forward: 5’ CCA AGT GCT GCC GTC ATT TTC 3’; reverse: 5’ GGC TCG CAG GGA TGA TTT CAA 3’), *Timp1* (forward: 5’ GCA ACT CGG ACC TGG TCA TAA 3’; reverse: 5’ CGG CCC GTG ATG AGA AAC T 3’), *Isg15* (forward: 5’ GGT GTC CGT GAC TAA CTC CAT 3’; reverse: 5’ TGG AAA GGG TAA GAC CGT CCT 3’), *Il1b* (forward: 5’ TCG AGG CCT AAT AGG CTC ATC T 3’; reverse: 5’ GCT GCT TCA GAC ACT TGC ACA A 3’), *Ifng* (forward: 5’ GCC ACG GCA CAG TCA TTG A 3’; reverse: 5’ TGC TGA TGG CCT GAT TGT CTT 3’), *Rsad2* (forward: 5’ TGC TGG CTG AGA ATA GCA TTA GG 3’; reverse: 5’ GCT GAG TGC TGT TCC CAT CT 3’), *Gzmb* (forward: 5’ CCA CTC TCG ACC CTA CAT GG 3’; reverse: 5’ GGC CCC CAA AGT GAC ATT TAT T 3’), *Il21* (forward: 5’ GGA CCC TTG TCT GTC TGG TAG 3’; reverse: 5’ TGT GGA GCT GAT AGA AGT TCA GG 3’), *Pdcd1* (forward: 5’ ACC CTG GTC ATT CAC TTG GG 3’; reverse: 5’ CAT TTG CTC CCT CTG ACA CTG 3’), *Cd3e* (forward: 5’ TCA GCC TCC TAG CTG TTG G 3’; reverse: 5’ GTC AAC TCT ACA CTG GTT CCT G 3’), *Cd4* (forward: 5’ AGG TGA TGG GAC CTA CCT CTC 3’; reverse: 5’ GGG GCC ACC ACT TGA ACT AC 3’), *Cd8a* (forward: 5’ CCG TTG ACC CGC TTT CTG T 3’; reverse: 5’ CGG CGT CCA TTT TCT TTG GAA 3’), *Cd8b1* (forward: 5’ CTC TGC CCT CAT TCA GAC CC 3’; reverse: 5’ AGA TGC TTT TAA CCT CAC AGG AC 3’), *Cd19* (forward: 5’ CTT GGT ATC GAG GTA ACC AGT CA 3’; reverse: 5’ ACA ATC ACT AGC AAG ATG CCC 3’), *Cd79b* (forward: 5’ CCG AGG TTT GCA GCC AAA AAG 3’; reverse: 5’ CAC AAT GCG TCC CTC TTC TG 3’), and *Ncr1* (forward: 5’ ATG CTG CCA ACA CTC ACT G 3’; reverse: 5’ GAT GTT CAC CGA GTT TCC ATT TG 3’).

### Detection and quantification of ZIKV RNA by RT-qPCR

ZIKV genomic RNA copy numbers were quantified as previously described^93^ with minor modifications. Briefly, the standard RNA of ZIKV was synthesized using the MEGAscript™ T7 Transcription Kit (Invitrogen) according to the manufacturer’s in vitro transcription procedure, using a DNA template representing nt 3442–3701 region of ZIKV NS1-NS2A gene, which was designed to be linked to the T7 promoter at the 5’-end. Total RNA was extracted, reverse-transcribed to cDNA, and qRT-PCR was performed using the following primers: forward 5′-GTATGGAATGGAGATAAGGCCCA-3′ and reverse 5′-GCACATCAATGGCAGTGCTGGT-3′. Calculation of the ZIKV RNA copy number based on the standard RNA was analyzed on the QuantStudio™ 3 Real-Time PCR System (Applied Biosystems). For comparative analyses between juvenile and adult brain samples, all qRT-PCR reactions were performed simultaneously on the same 96-well plate using identical reaction conditions, and viral RNA copy numbers were calculated using a shared standard curve to ensure cross-sample comparability.

### Statistical analysis

All statistical analyses were performed using GraphPad Prism. Experiments were conducted independently at least twice. Differences between groups were analyzed using a two-tailed Student’s *t* test with unequal variance. *P* < 0.05 was considered statistically significant. Data are presented as mean ± SEM unless otherwise specified.

## Supplementary Information

Below is the link to the electronic supplementary material.

Supplementary Material 1

Supplementary Material 2

Supplementary Material 3

Supplementary Material 4

Supplementary Material 5

Supplementary Material 6

Supplementary Material 7

Supplementary Material 8

Supplementary Material 9

Supplementary Material 10

Supplementary Material 11

## Data Availability

All data generated or analyzed during this study are included in this published article and its supplementary information files.
