# Supplementary figures and images for: Brain-infiltrating CD8 T cells retain functional activity to protect against acute Zika virus infection
Source: Sci Rep. 2026 Jan 5;16:4738. doi: 10.1038/s41598-026-35079-3 (PMC12873414; doi:10.1038/s41598-026-35079-3)

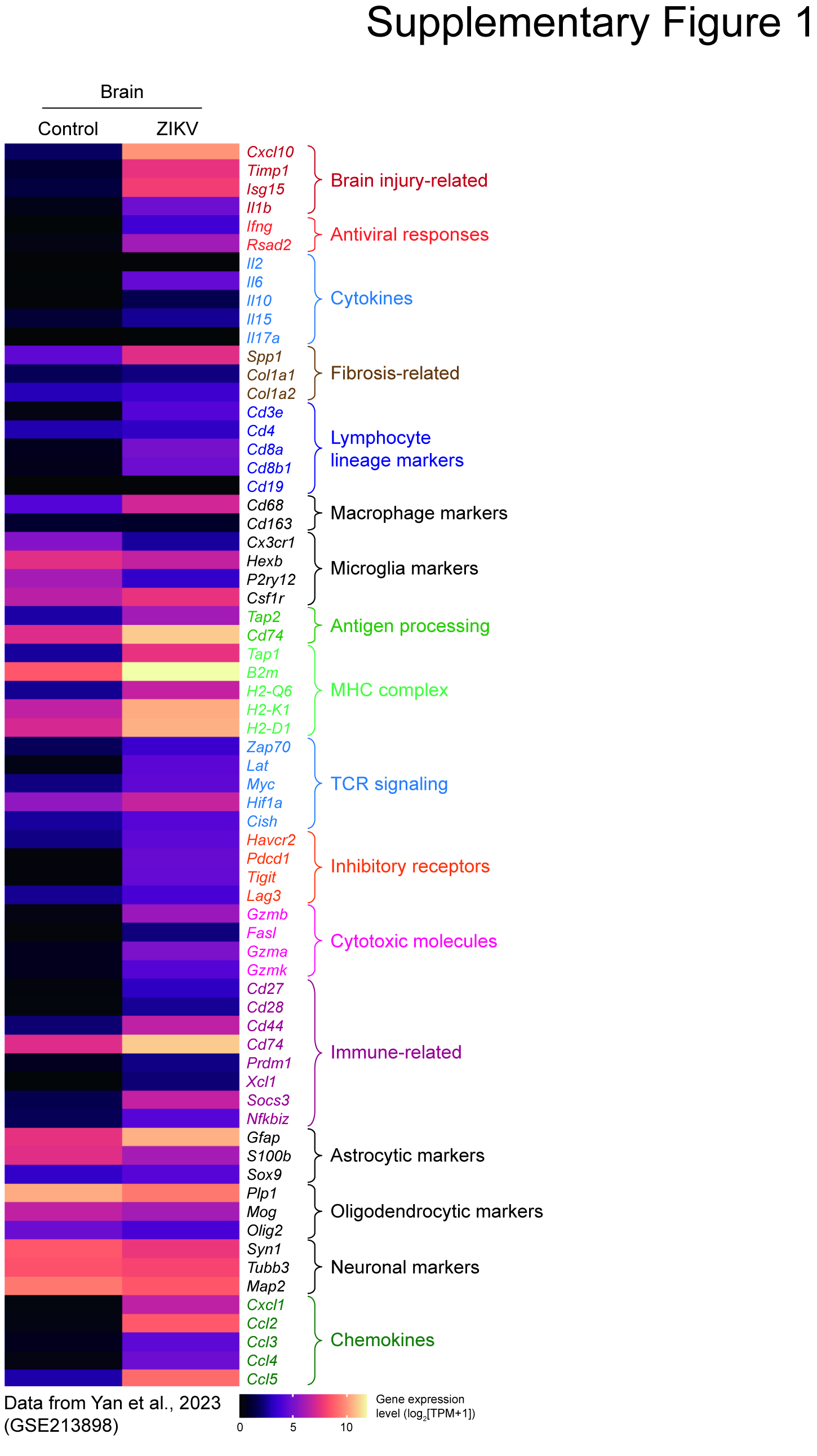

Supplement: Supplementary file 1 — Supplementary Material 1 [file 41598_2026_35079_MOESM1_ESM.tif]

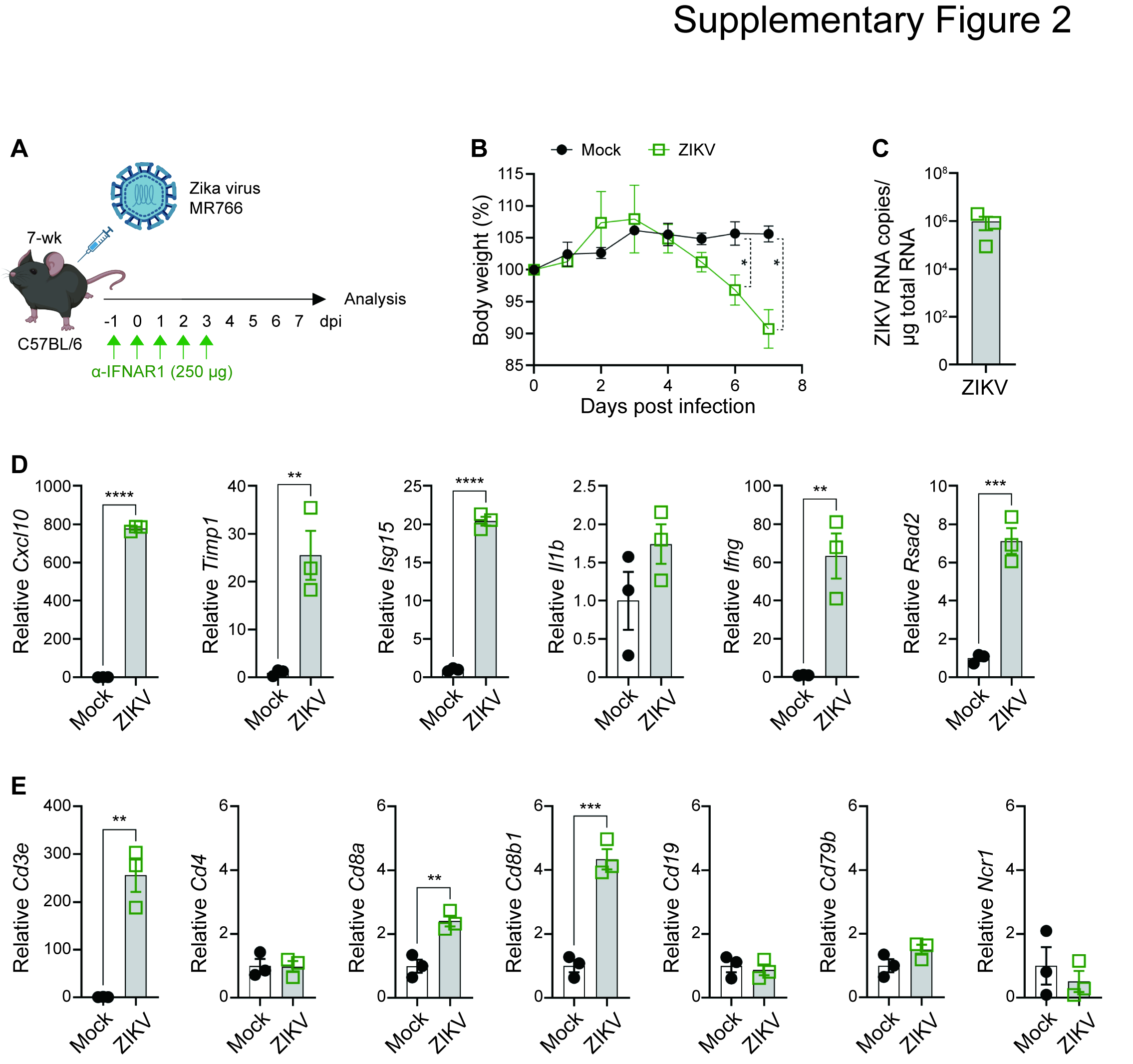

Supplement: Supplementary file 2 — Supplementary Material 2 [file 41598_2026_35079_MOESM2_ESM.tif]

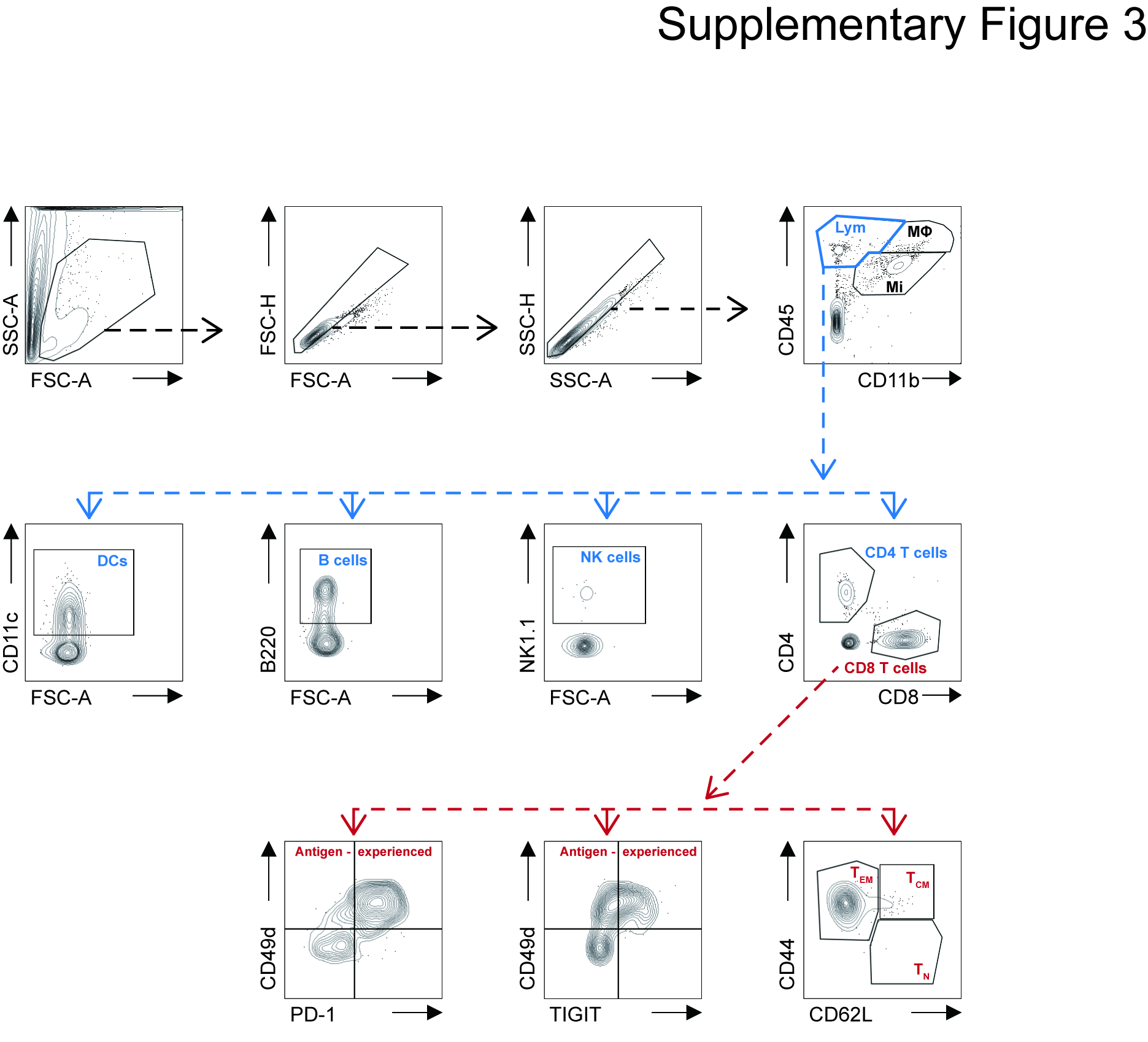

Supplement: Supplementary file 3 — Supplementary Material 3 [file 41598_2026_35079_MOESM3_ESM.tif]

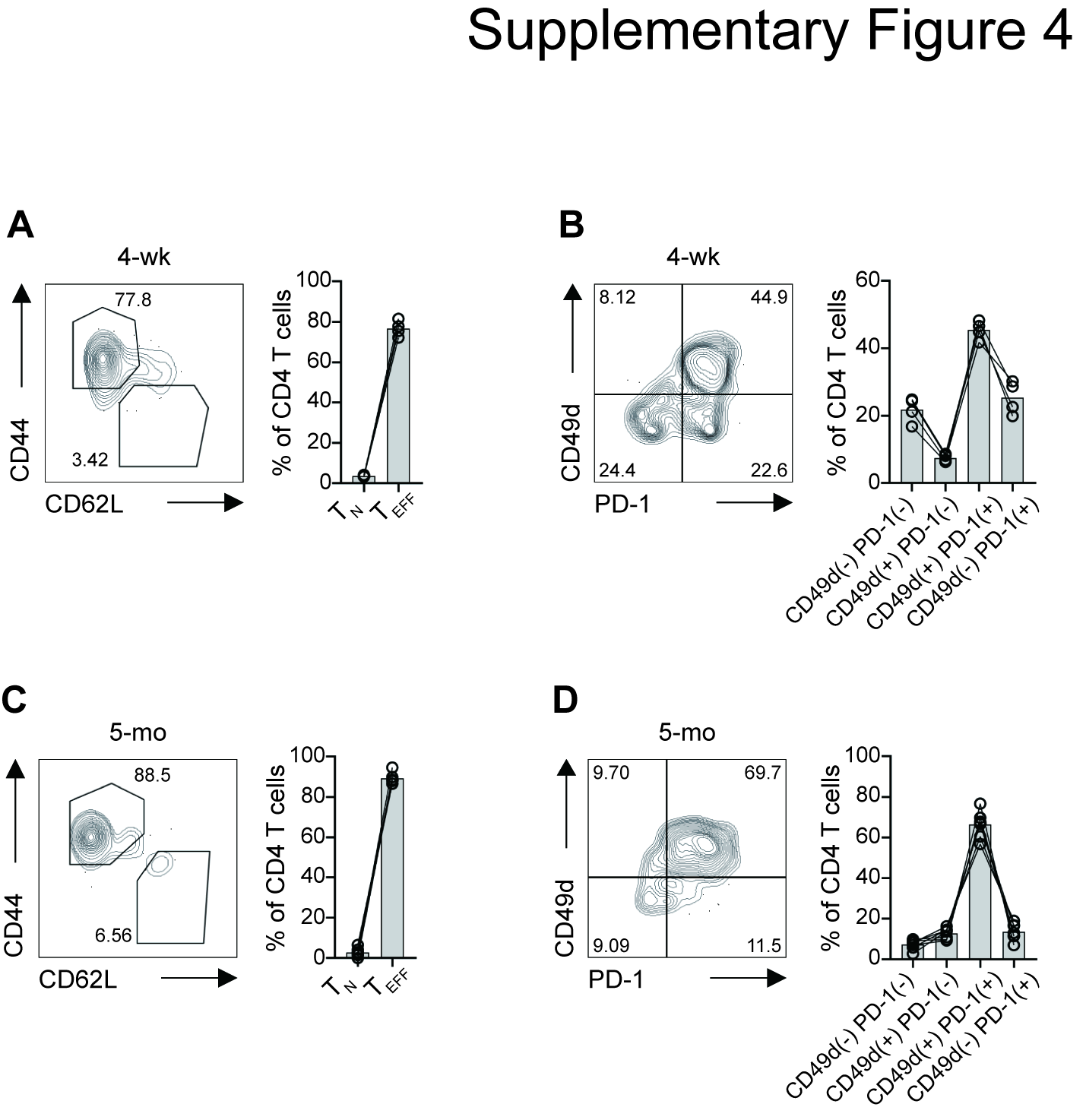

Supplement: Supplementary file 4 — Supplementary Material 4 [file 41598_2026_35079_MOESM4_ESM.tif]

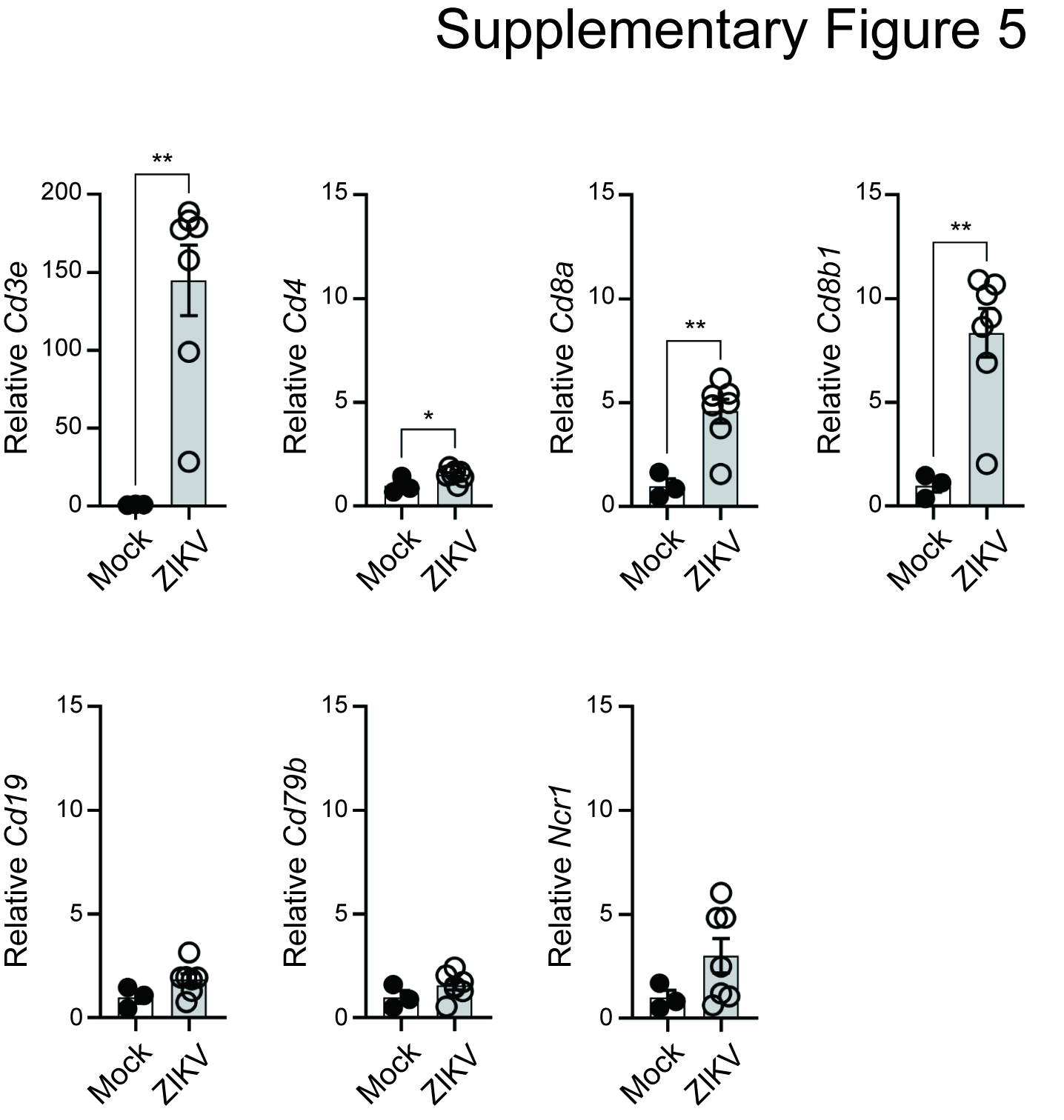

Supplement: Supplementary file 5 — Supplementary Material 5 [file 41598_2026_35079_MOESM5_ESM.tif]

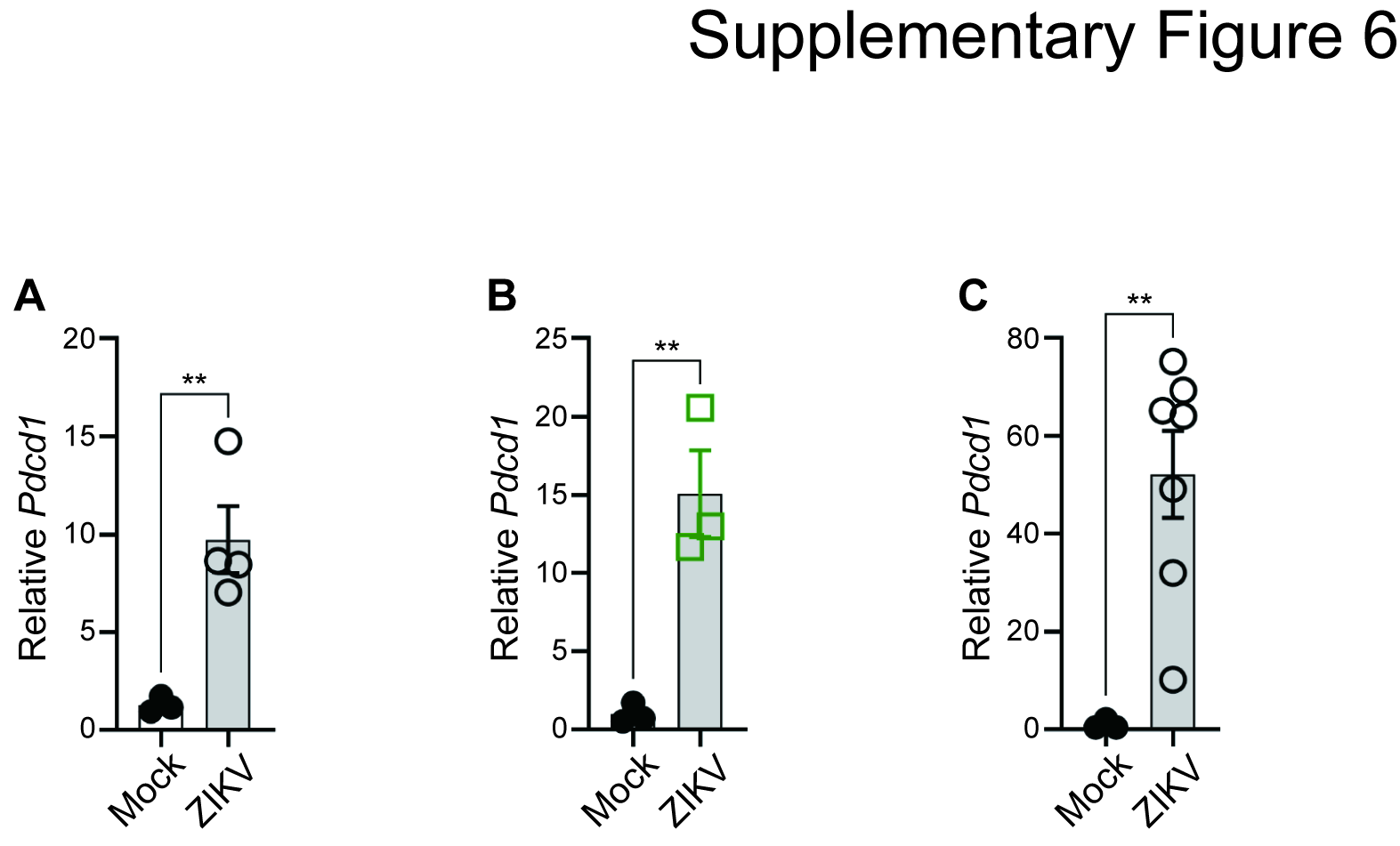

Supplement: Supplementary file 6 — Supplementary Material 6 [file 41598_2026_35079_MOESM6_ESM.tif]

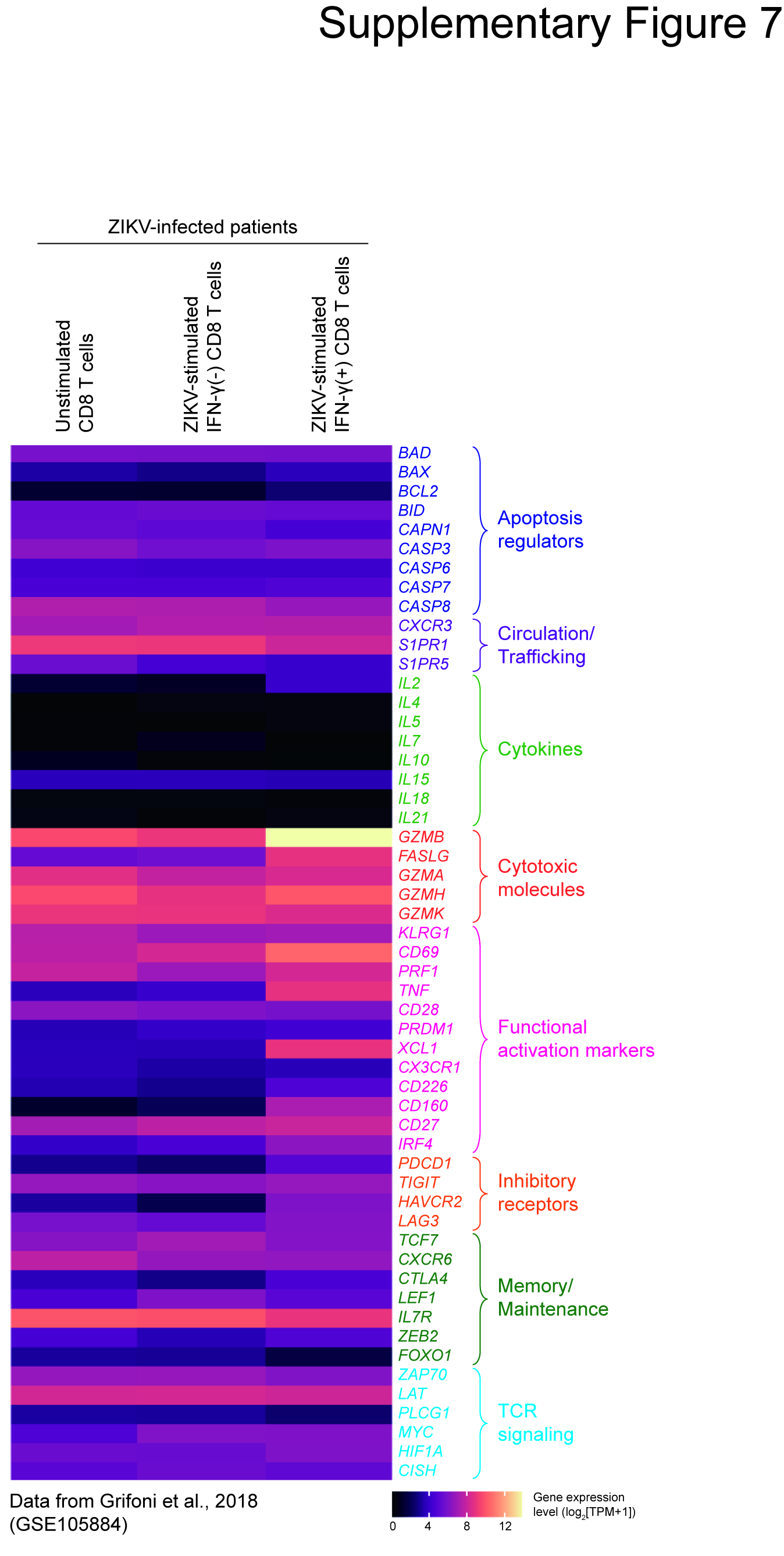

Supplement: Supplementary file 7 — Supplementary Material 7 [file 41598_2026_35079_MOESM7_ESM.tif]

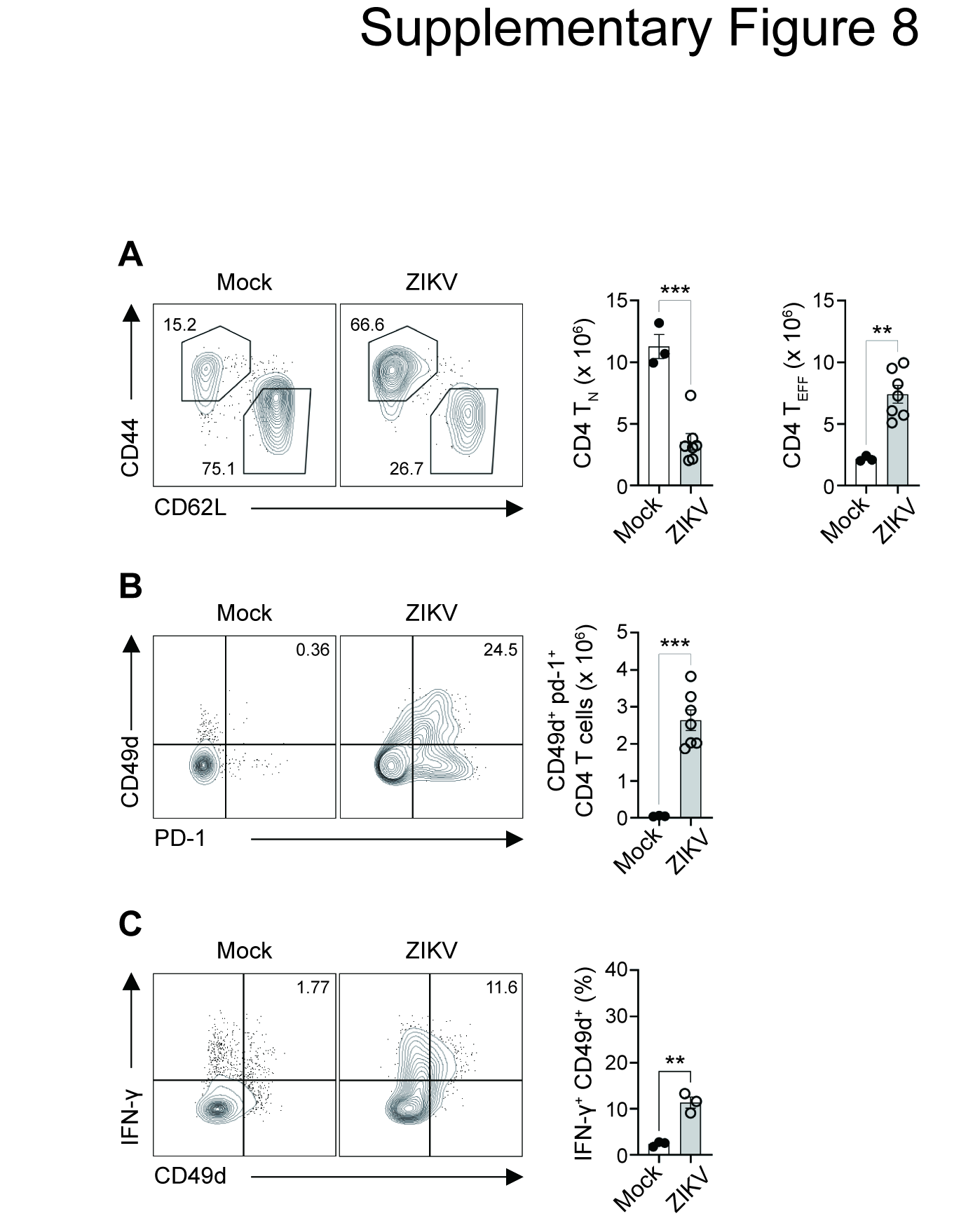

Supplement: Supplementary file 8 — Supplementary Material 8 [file 41598_2026_35079_MOESM8_ESM.tif]

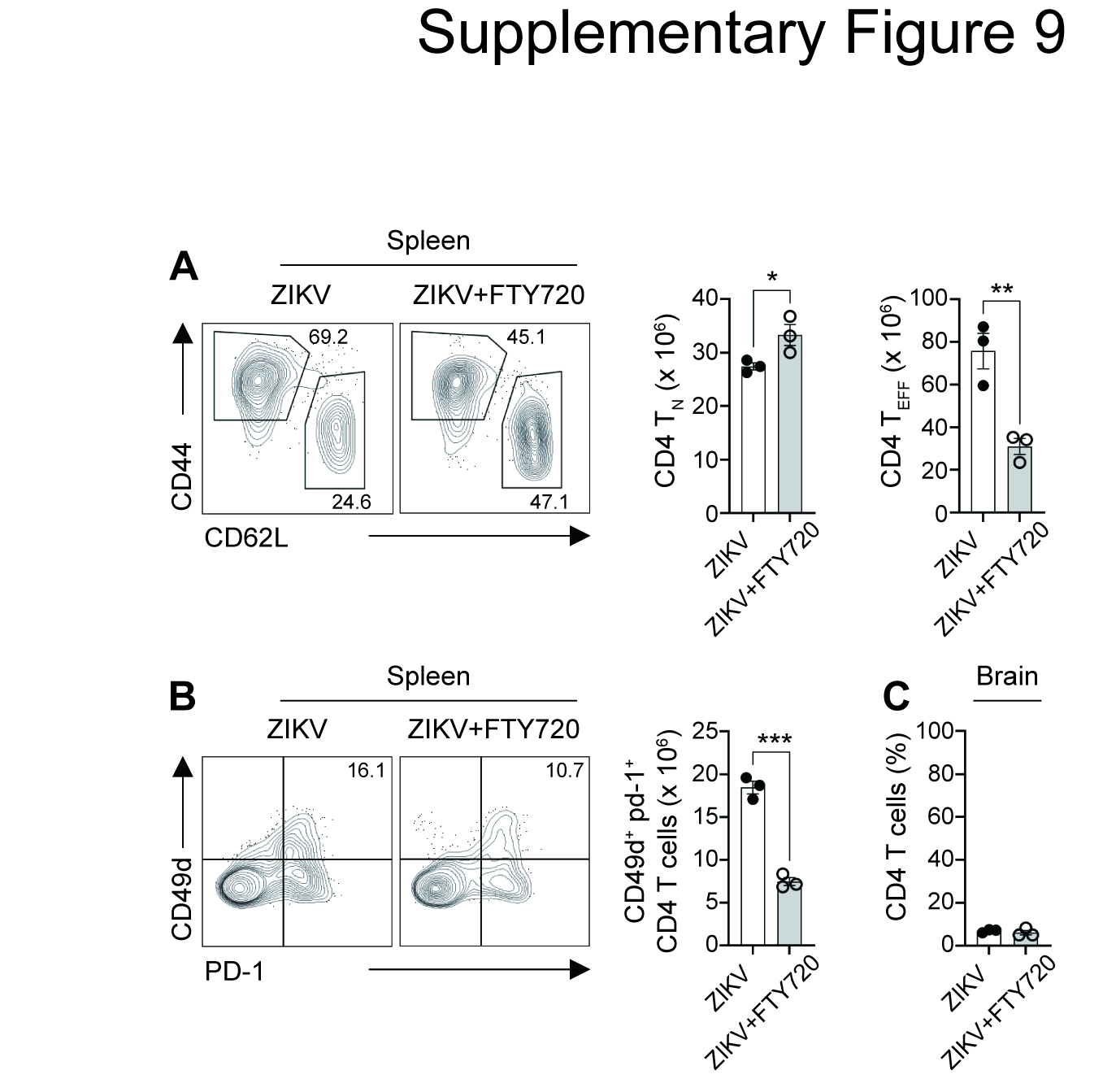

Supplement: Supplementary file 9 — Supplementary Material 9 [file 41598_2026_35079_MOESM9_ESM.tif]

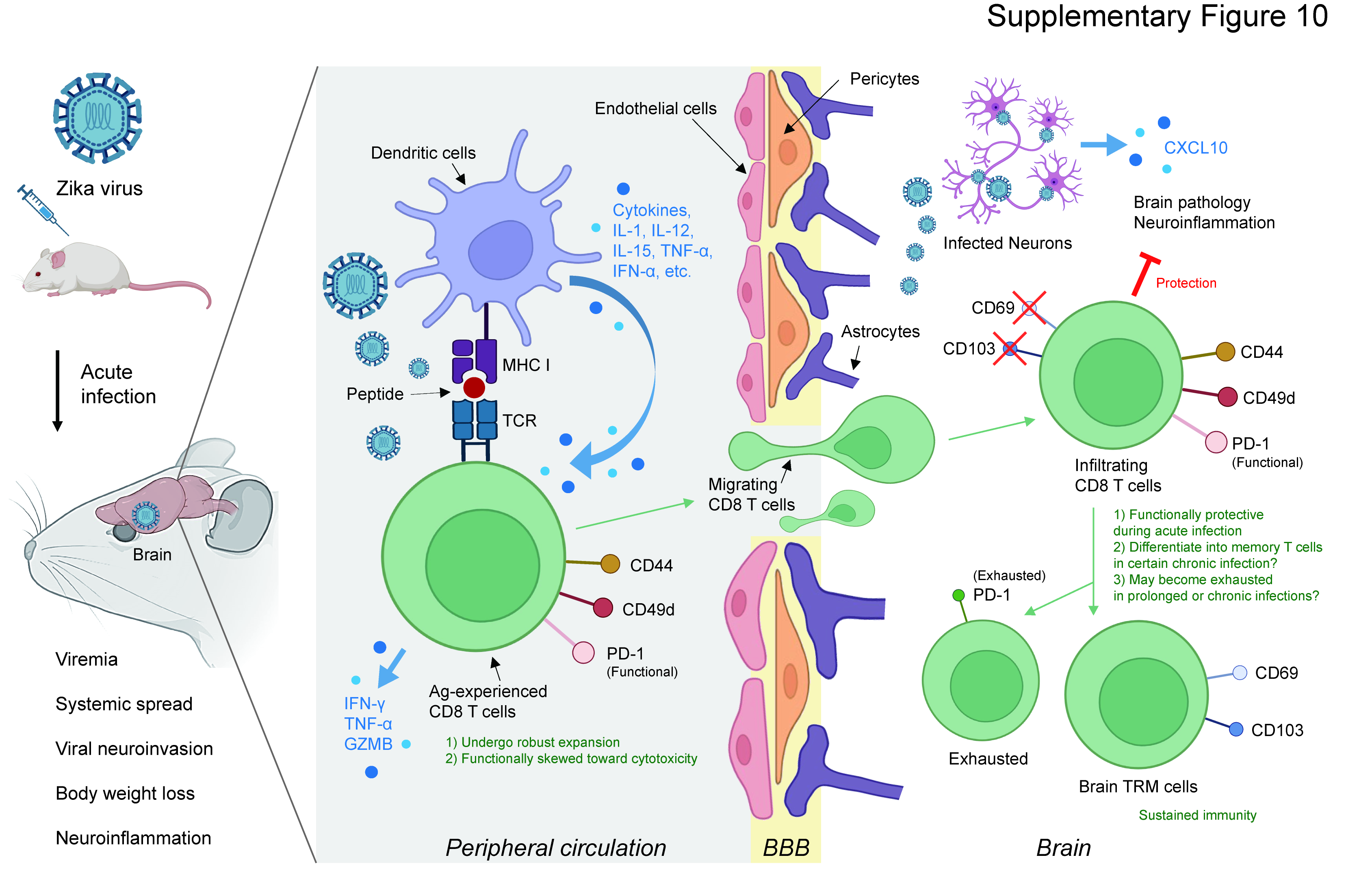

Supplement: Supplementary file 10 — Supplementary Material 10 [file 41598_2026_35079_MOESM10_ESM.tif]
