# Supplementary material for: Brain-infiltrating CD8 T cells retain functional activity to protect against acute Zika virus infection
Source: Sci Rep. 2026 Jan 5;16:4738. doi: 10.1038/s41598-026-35079-3 (PMC12873414; doi:10.1038/s41598-026-35079-3)
Supplement: Supplementary file 11 — Supplementary Material 11 [file 41598_2026_35079_MOESM11_ESM.docx]

**Supplementary Figure Legends**

**Supplementary Figure 1. Transcriptomic profiling of brain tissue from ZIKV-infected and control mice (data from Yan *et al.*, 2023; GSE213898).**

Heatmap of gene expression in brain tissue from ZIKV-infected and control mice, grouped by functional categories. Mice were observed daily for clinical signs and euthanized on day 9 postinfection prior to reaching humane endpoints. Brain tissue was collected for RNA-seq analysis. ZIKV-infected (*n* = 3) and control (*n* = 3) brain samples were analyzed. Genes are organized into categories including brain injury–related, antiviral responses, cytokines, fibrosis-related, lymphocyte lineage markers, macrophage markers, microglia markers, antigen processing, MHC complex, TCR signaling, inhibitory receptors, cytotoxic molecules, immune-related, astrocytic markers, oligodendrocytic markers, neuronal markers, and chemokines. Expression values were calculated as TPM, log_2_-transformed [log_2_(TPM+1)], and averaged across biological replicates for each group. Data from Yan *et al.*, 2023 (*J. Med. Virol.*); GSE213898. Figure generated by reanalysis and replotting of the publicly available dataset.

**Supplementary Figure 2. ZIKV infection is associated with brain pathology in α-IFNAR1-treated C57BL/6 mice.**

(A-D) Seven-week-old C57BL/6 (B6) mice were infected with the ZIKV MR766 strain. To enhance susceptibility to infection, α-IFNAR1 antibody was administered on days -1, 0, 1, 2, and 3 post-infection. (A) Schematic illustration of the experimental mouse model. (B) Daily body weight changes following ZIKV infection. Error bars indicate SEM. (C) qRT-PCR analysis of ZIKV RNA levels in the brain at 7 days post-infection. (D) qRT-PCR analysis of neuronal injury-related and antiviral immune responses in the brain at 7 days post-infection. (E) qRT-PCR analysis of lymphocyte lineage marker transcripts in the brain at 7 days post-infection. Data are presented as mean ± SEM. ***P*<0.01; ****P*<0.005; *****P*<0.001 (two-tailed two-sample unequal variance Student *t* test).

**Supplementary Figure 3. Gating strategy for immune cell identification using flow cytometry.**

A sequential gating strategy was used to identify immune cell populations in the brains of *Ifnar1*^-^/^-^ mice following ZIKV infection. Representative flow cytometry plots illustrate the gating process for lymphocytes (Lym; CD45^hi^ CD11b^-^), macrophages (MΦ; CD45^hi^ CD11b^+^), microglia (Mi; CD45^int^ CD11b^+^), dendritic cells (DCs), B cells, NK cells, CD4⁺ T cells, and CD8⁺ T cells. Additional gating was applied to define CD8^+^ T cell subsets based on the expression of CD44, CD62L, CD49d, PD-1, and TIGIT.

**Supplementary Figure 4. Characteristics of CD4^+^ T cells in ZIKV-infected juvenile and mature adult mice.**

(A-B) Four-week-old *Ifnar1*^-^/^-^ mice were infected with ZIKV. Five days post-infection, brains were harvested and CD4^+^ T cells were analyzed. (A) Representative FACS plots (left) and bar graphs (right) depicting the frequencies of CD44^lo^ CD62L^hi^ naïve CD4^+^ T cells (T_N_) and CD44^hi^ CD62l^lo^ effector memory CD4^+^ T cells (T_EFF_) in the brains of ZIKV-infected mice. (B) CD4^+^ T cells were analyzed based on the expression of CD49d and PD-1. Representative FACS plots (left) and bar graphs (right) are shown. (C-D) Five-month-old *Ifnar1*^-^/^-^ mice were infected with ZIKV. Six days post-infection, brain-infiltrating CD4^+^ T cells were analyzed for the expression of CD44 and CD62L (C) and for the expression of CD49d and PD-1 (D). Data are representative of two independent experiments. All data are presented as mean ± SEM. **P*<0.05; ***P*<0.01; ****P*<0.005; *****P*<0.001 (two-tailed two-sample unequal variance Student *t* test).

**Supplementary Figure 5. RT-qPCR analysis of lymphocyte lineage markers in the brain.**

Five-month-old *Ifnar1*^-^/^-^ mice were infected with ZIKV, and expression of lymphocyte lineage–associated transcripts in the brain was quantified at 6 days post-infection. Data represent two independent experiments and are presented as mean ± SEM. **P*<0.05; ***P*<0.01 (two-tailed two-sample unequal variance Student *t* test).

**Supplementary Figure 6. Increased expression of *Pdcd1* in the brains of ZIKV-infected mice.**

(A) qRT-PCR analysis of *Pdcd1* expression in the brains of ZIKV-infected 4-week-old *Ifnar1*^-^/^-^ mice at 5 days post-infection. (B) qRT-PCR analysis of *Pdcd1* expression in the brains of ZIKV-infected 7-week-old C57BL/6 mice treated with α-IFNAR1 at 7 days post-infection. (C) qRT-PCR analysis of *Pdcd1* expression in the brains of ZIKV-infected 5-month-old *Ifnar1*^-^/^-^ mice at 6 days post-infection. Data are presented as mean ± SEM. ***P*<0.01 (two-tailed two-sample unequal variance Student *t* test).

**Supplementary Figure 7. Transcriptomic profile of ZIKV-stimulated and unstimulated CD8 T cell subsets from PBMCs of ZIKV-infected donors (data from Grifoni *et al.*, 2018; GSE105884).**

Heatmap of gene expression across functional categories, including apoptosis regulators, trafficking molecules, cytokines, cytotoxic effectors, activation markers, immune checkpoint receptors, memory/maintenance markers, and TCR signaling molecules. PBMCs collected at the convalescent phase from ZIKV-infected donors were stimulated *ex vivo* with ZIKV peptide pools, and antigen-responsive IFN-γ^+^ CD8^+^ T cells, non–IFN-γ–producing CD8^+^ T cells from the same cultures, and total unstimulated CD8^+^ T cells were sorted for transcriptomic analysis. Expression values were calculated as TPM, log_2_-transformed [log_2_(TPM+1)], and averaged across biological replicates for each group. Data from Grifoni *et al.*, 2018 (*J. Immunol.*); GSE105884. Figure generated by reanalysis and replotting of the publicly available dataset.

**Supplementary Figure 8. Expansion of ZIKV-experienced CD4^+^ T cells in the spleen.**

(A-C) Five-month-old *Ifnar1*^-^/^-^ mice were infected with ZIKV, and spleens were harvested at 6 days post-infection. (A) Representative FACS plots (left) and bar graphs (right) depicting the frequencies of T_N_ and T_EFF_ in the spleens of mock and ZIKV-infected mice. (B) Representative FACS plots (left) and bar graphs (right) showing the frequencies of CD49d^+^ PD-1^+^ CD4^+^ T cells in the spleens of *Ifnar1*^-^/^-^ mice. (C) Splenocytes from mock and ZIKV-infected mice were stimulated with PMA and ionomycin in the presence of GolgiStop and GolgiPlug for 5 hours, and the frequency of IFN-γ-expressing CD49d^+^ CD4^+^ T cells was assessed. Data are representative of two independent experiments. All data are presented as mean ± SEM. ***P*<0.01; ****P*<0.005 (two-tailed two-sample unequal variance Student *t* test).

**Supplementary Figure 9. Effect of FTY720 on brain-infiltrating CD4^+^ T cells.**

(A-C) Five-month-old *Ifnar1*^-^/^-^ mice were infected with the ZIKV PRVABC59 strain, and FTY720 was administered on days 0, 1, 4, and 5 post-infection. At six days post-infection, spleen and brain tissues were harvested for analysis. (A) Representative FACS plots (left) and bar graphs (right) showing the absolute numbers of T_N_ and T_EFF_ CD4^+^ T cells in the spleen of ZIKV-infected mice with or without FTY720 treatment. (B) Representative FACS plots (left) and bar graphs (right) depicting the absolute numbers of CD49d^+^ PD-1^+^ CD4^+^ T cells in the spleens of ZIKV-infected mice. (C) Frequency of CD4^+^ T cells within the brain lymphocyte gate (CD45^hi^ CD11b^-^). Data are representative of two independent experiments. All data are presented as mean ± SEM. **P*<0.05; ***P*<0.01; ****P*<0.005 (two-tailed two-sample unequal variance Student *t* test).

**Supplementary Figure 10. Schematic summary of CD8^+^ T cell dynamics in ZIKV infection.**

Following acute infection, viral dissemination and neuroinvasion are associated with brain pathology and neuroinflammation. Dendritic cells activate CD8^+^ T cells in the periphery through antigen presentation, driving robust expansion and functional skewing toward cytotoxicity. Activated, antigen-experienced CD8^+^ T cells (CD44^+^, CD49d^+^, PD-1^+^) migrate across the BBB and infiltrate the brain, where they exert protective antiviral effects. Infiltrating CD8^+^ T cells may remain functionally active, differentiate into brain T_RM_ cells that sustain long-term immunity, or become exhausted during prolonged or chronic infection.
